# Genetic background variation impacts microglial heterogeneity and disease progression in amyotrophic lateral sclerosis model mice

**DOI:** 10.1016/j.isci.2024.108872

**Published:** 2024-01-11

**Authors:** Okiru Komine, Syuhei Ohnuma, Kunihiko Hinohara, Yuichiro Hara, Mayuko Shimada, Tomohiro Akashi, Seiji Watanabe, Akira Sobue, Noe Kawade, Tomoo Ogi, Koji Yamanaka

**Affiliations:** 1 Department of Neuroscience and Pathobiology, Research Institute of Environmental Medicine, Nagoya University, Nagoya, Aichi, Japan; 2 Department of Neuroscience and Pathobiology, Nagoya University Graduate School of Medicine, Nagoya University, Nagoya, Aichi, Japan; 3 Department of Immunology, Nagoya University Graduate School of Medicine, Nagoya, Aichi, Japan; 4 Institute for Advanced Research, Nagoya University, Nagoya, Aichi, Japan; 5 Center for 5D Cell Dynamics, Nagoya University, Nagoya, Aichi, Japan; 6 Department of Genetics, Research Institute of Environmental Medicine, Nagoya University, Nagoya, Aichi, Japan; 7 Department of Human Genetics and Molecular Biology, Nagoya University Graduate School of Medicine, Nagoya, Aichi, Japan; 8 Center for Neurological Disease and Cancer, Nagoya University Graduate School of Medicine, Nagoya, Aichi, Japan; 9 Medical Interactive Research and Academia Industry Collaboration Center, Research Institute of Environmental Medicine, Nagoya University, Aichi, Japan; 10 Institute for Glyco-core Research (iGCORE), Nagoya University, Aichi, Japan; 11 Center for One Medicine Innovative Translational Research (COMIT), Nagoya University, Aichi, Japan

**Keywords:** Genetics, Neuroscience, Immunology

## Abstract

Recent single-cell analyses have revealed the complexity of microglial heterogeneity in brain development, aging, and neurodegenerative diseases such as amyotrophic lateral sclerosis (ALS). Disease-associated microglia (DAMs) have been identified in ALS mice model, but their role in ALS pathology remains unclear. The effect of genetic background variations on microglial heterogeneity and functions remains unknown. Herein, we established and analyzed two mice models of ALS with distinct genetic backgrounds of C57BL/6 and BALB/c. We observed that the change in genetic background from C57BL/6 to BALB/c affected microglial heterogeneity and ALS pathology and its progression, likely due to the defective induction of neurotrophic factor-secreting DAMs and impaired microglial survival. Single-cell analyses of ALS mice revealed new markers for each microglial subtype and a possible association between microglial heterogeneity and systemic immune environments. Thus, we highlighted the role of microglia in ALS pathology and importance of genetic background variations in modulating microglial functions.

## Highlights

• G93AL(Balb) shows faster disease progression with distinct microglial heterogeneity
• G93AL(Balb) microglia displays lower productions of IGF-1, M-CSF, and chemokines
• G93AL(Balb) exhibits a defective induction of *Igf1*-expressing DAM cluster
• Genetic diversity may affect microglial heterogeneity and responses

## Graphical abstract

**Figure:**
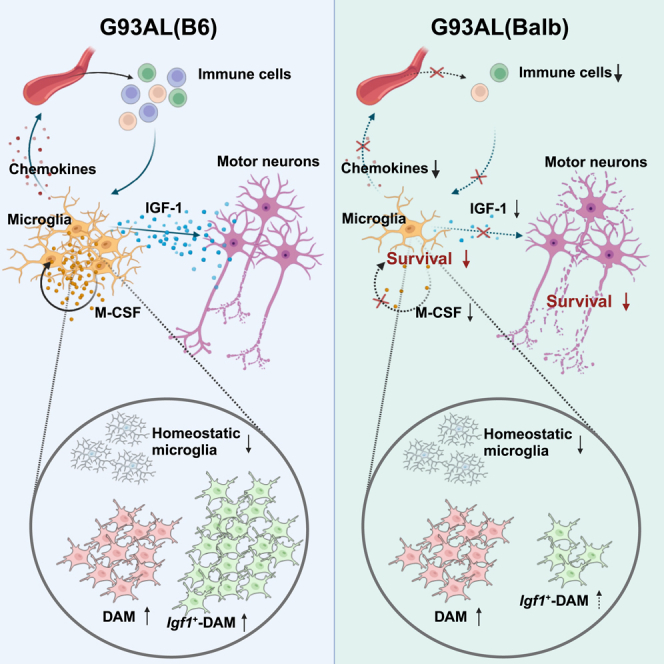

## Introduction

Recent advances in single-cell RNA sequencing (scRNA-seq) technologies have revealed the intrinsic heterogeneity of microglia, which are resident innate immune cells in the central nervous system (CNS) derived from the yolk sac, and their role in maintaining homeostasis under healthy conditions.^1^^,^^2^ However, microglial gene expression profiles become heterogeneously altered under disease conditions in response to neuronal damage.^3^^,^^4^^,^^5^ Recent studies using RNA-sequencing and scRNA-seq identified unique a subpopulation of activated microglia termed as Disease-associated microglia (DAM), Microglial neurodegenerative phenotype (MGnD), and Activated response microglia (ARM) that have been closely associated with amyloid-β plaques in the cerebral cortices of the model mice for Alzheimer’s disease (AD), the most common cause of dementia, and have characteristic features of the gene expression profiles.^3^^,^^4^^,^^5^ DAMs/MGnDs have also been identified in model mice for amyotrophic lateral sclerosis (ALS), an adult motor neuron disease, expressing a mutant form of superoxide dismutase 1 (SOD1).^3^^,^^4^ Although the expression of DAM-related genes has been shown to increase with the reduced expression of homeostatic microglial genes in isolated microglia from both AD and ALS model mice,^6^ the exact contributions of DAM to pathological processes in these diseases remain unknown. Intriguingly, natural genetic variation has been shown to affect microglial heterogeneity in wild-derived mouse models of AD.^7^ In particular, WSB/EiJ-derived AD model mice did not exhibit an increase in DAM or a decrease in homeostatic microglia. Notably, although SOD1-expressing ALS model mice with different genetic backgrounds exhibit different disease time courses,^8^^,^^9^^,^^10^^,^^11^^,^^12^^,^^13^^,^^14^ there are no studies revealing the mechanism. Moreover, whether genetic background variation affects microglial heterogeneity and functions in ALS remains unclear.

The role of microglia in ALS has been extensively studied, but their function remains debatable due to their double-edged sword nature^15^ and the complexity of heterogeneity. Infiltrating T-lymphocytes can regulate the balance between the beneficial and detrimental functions of microglia by inducing the secretion of insulin-like growth factor 1 (IGF-1), a neurotrophic factor.^16^ In addition, the upregulated expression levels of genes including *Igf1* and DAM-related genes in microglia could correlate with the infiltration ratio of T-lymphocytes in mutant SOD1-expressing ALS model mice.^17^ Recent scRNA-seq analysis revealed the predominance of *Igf1* expression in DAM.^3^^,^^18^ Furthermore, alterations in the peripheral immune system (in both immune cell populations and functions) have been reported in patients with ALS and in the ALS mouse model.^19^ Therefore, the peripheral immune environment may affect microglial heterogeneity and the disease course in ALS, but whether this environment affects microglia or immune cells in the CNS and ALS progression remains unclear.

This study explored the effect of genetic background variations on microglial heterogeneity and functions in ALS model mice. Following the generation of two kinds of SOD1^G93A^ ALS model mice with different genetic backgrounds, C57BL/6 and BALB/c strains, as well as wild-type littermates, single-cell RNA-seq analysis was performed in the spinal cords of the mice, followed by survival analysis. We established for the first time that the genetic change from C57BL/6 to BALB/c background affects microglial heterogeneity and ALS pathology, as evidenced by the reduced induction of DAM clusters, fewer surviving microglia, and accelerated disease progression in ALS mice. In addition, a neuroprotective microglial subtype was identified in these DAM clusters. Our findings suggest that environmental factors derived from peripheral immune cells may contribute to the phenotypic differences observed in microglia.

## Results

### Single-cell RNA sequencing analysis revealed that genetic background variation affected microglial heterogeneity in amyotrophic lateral sclerosis model mice

SOD1^G93A^ mice carrying a low copy number of mutant SOD1 in the C57BL/6J background (G93AL(B6)) were used, which spontaneously lost transgene copies (∼−40%) during laboratory breeding. G93AL(B6) mice were backcrossed over 10 times with BALB/cA mice to generate congenic mice (G93AL(Balb)), and quantitative PCR (qPCR) was used to confirm the comparability of the transgene copy number for each mouse.

**Figure 1:**
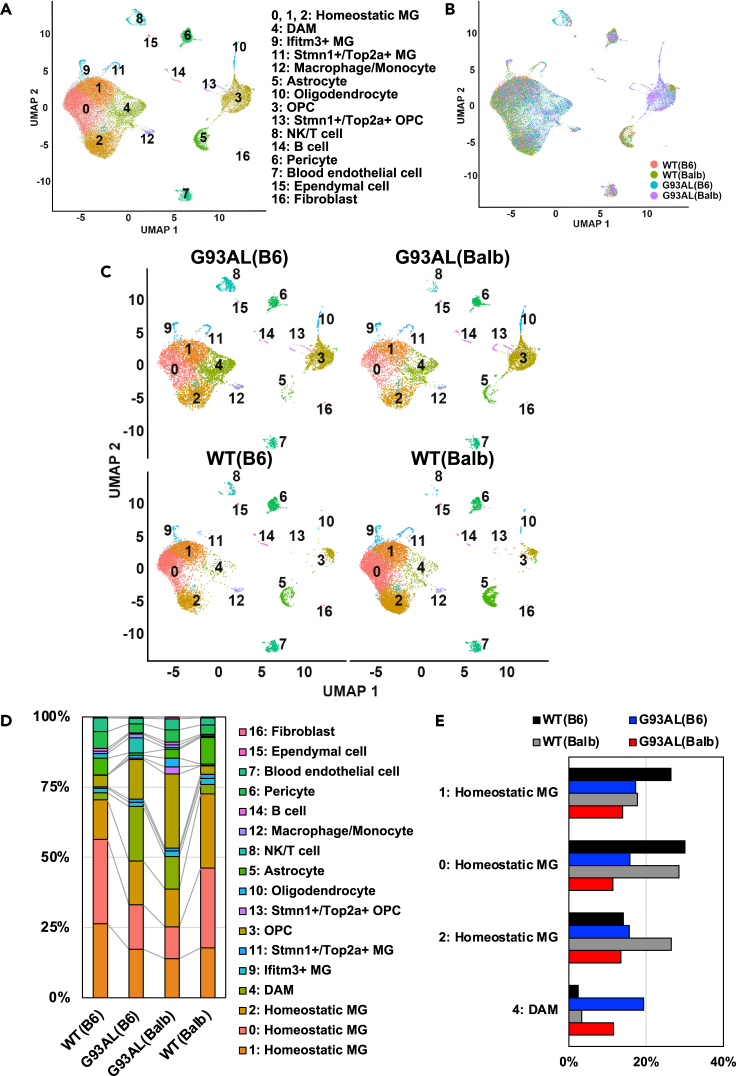
Genetic background variation affects microglial heterogeneity in ALS model mice (A–C) All single-cell transcriptome data (31,216 cells) of UMAP plots for the spinal cords of ALS model mice at the disease end-stage and age-matched litter mate wild-type mice (6,473 cells from WT(B6), 9,762 cells from WT(Balb), 8,391 cells from G93AL(B6), and 6,590 cells from G93AL(Balb)). Each cluster was colored by cell type (A and C) or mouse strain (B). (D) Percentages of each cell type in the single-cell transcriptome plots from each strain were plotted. (E) Percentages of each homeostatic microglia cluster and DAM cluster from each strain were plotted.

To examine the effect of genetic background variation on microglial heterogeneity under healthy and ALS conditions, scRNA-seq analysis was performed in the spinal cords of G93AL(B6), G93AL(Balb), and both wild-type (WT(B6) and WT(Balb)) mice at the disease end-stage. Clustering analysis showed that these samples were separated into 17 clusters: homeostatic microglia (MG) 0, 1, and 2, DAM, Ifitm3+ (interferon response microglia) MG, Stmn1+/Top2a+ (proliferating microglia) MG, macrophage/monocyte, astrocyte, oligodendrocyte, oligodendrocyte progenitor cell (OPC), Stmn1+/Top2a+ OPC (proliferating OPC), natural killer (NK)/T cell, B cell, pericyte, blood endothelial cell, ependymal cell, and fibroblast, with the cell-type-specific marker gene expressions (Figures 1A, 1B, S1A, and S1B). In particular, microglia subpopulations were characterized into six clusters, similar to previous studies.^1^^,^^3^^,^^5^^,^^7^ Unfortunately, not only all neurons but also most oligodendrocytes and astrocytes were lost during the myelin removal step in our method since these cells tightly attach. Although most microglia comprised the homeostatic MG clusters (clusters 0, 1, and 2) in both strains of wild-type mice, the ratios of clusters 1 and 2 differed (Figures 1A−1E). Notably, while G93AL(B6) microglia were found to exhibit an increased ratio of DAM with decreasing ratios of homeostatic MG clusters 0 and 1, G93AL(Balb) microglia displayed a slightly increased ratio of DAM with decreasing ratios of homeostatic MG clusters 0 and 2 (Figures 1C−1E). As previously reported,^20^^,^^21^^,^^22^ increased numbers of OPC were also observed in both strains of ALS mice (Figures 1C and 1D).

### Low-copy SOD1^G93A^ mice with the BALB/c background displayed faster disease progression with a decreasing number of microglia

**Figure 2:**
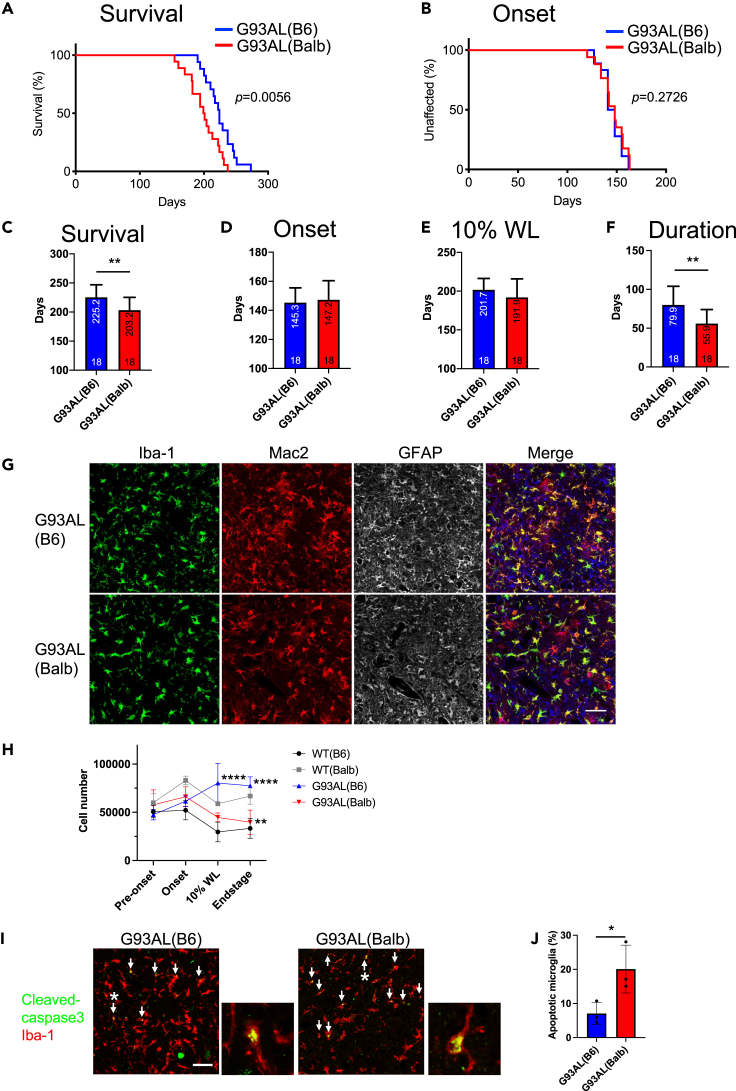
Low-copy SOD1^G93A^ mice with the BALB/c genetic background display faster disease progression with a decreased number of microglia (A) Survival times of low-copy SOD1^G93A^ mice with the C57BL/6 genetic background (G93AL(B6)) (blue, n = 18) and that with BALB/c one (G93AL(Balb)) (red, n = 18) were plotted in Kaplan–Meier curves. Statistical analysis of survival time was performed with the log rank test. (B) No difference in the ages of disease onset of SOD1^G93A^ mice with the indicated backgrounds. The onset times were plotted as Kaplan–Meier curves. The number of animals was the same as in (A). Statistical analysis of onset time was performed with the log rank test. (C) Plotted mean survival time of each strain showed shortened survival time of G93AL(Balb). Data were presented as mean ± SD. Unpaired *t*-test, ∗∗p *<* 0.01. (D and E) Plotted mean onset age (D) and mean age of 10% weight loss (E) showed no differences for each strain. Data were presented as mean ± SD. Unpaired t-tests. (F) Plotted mean disease duration of each strain showed shortened duration of G93AL(Balb) mice. Unpaired t-test, ∗∗p *<* 0.01. (G) Representative immunofluorescence images of the lumbar spinal cord sections of G93AL(B6) and G93AL(Balb) at the disease end-stage stained for Iba-1 (green), Mac2 (red), and GFAP (white or blue) along with the merged images. The number of G93AL(Balb) microglia was fewer than that of G93AL(B6) microglia, even though microglial activation was normally observed in both strains. Scale bar, 50 μm. (H) The numbers of CD45^low^ microglia isolated from the spinal cords were quantified by flow cytometric analysis at pre-onset (130 days of age (dA)), onset (145 dA), 10% body weight loss (10%WL) (WT(B6) and G93AL(B6): 200 dA, WT(Balb) and G93AL(Balb): 190 dA), and disease end-stage (WT(B6) and G93AL(B6): n = 3 each; WT(Balb): pre-onset, onset, and 10%WL, n = 3 each, end-stage, n = 4; G93AL(Balb): pre-onset and 10%WL, n = 3 each, onset, n = 5, end-stage, n = 4). In comparison to WT mice, the number of microglia was gradually increased in G93AL(B6) after onset (10%WL, p *<* 0.0001; end-stage, p *<* 0.0001), whereas that was gradually decreased in G93AL(Balb) after onset (end-stage, p *<* 0.01). Data were represented as mean ± SD. two-way ANOVA followed by Tukey–Kramer multiple comparison post hoc tests at each time point. ∗∗p *<* 0.01, ∗∗∗p *<* 0.001, ∗∗∗∗p *<* 0.0001. (I) Representative images of double-immunofluorescence staining for cleaved caspase 3 (green) and Iba-1 (red) in the lumbar spinal cord of G93AL(B6) and G93AL(Balb) at the disease end-stage. Arrows indicate cleaved caspase 3-positive (apoptotic) microglia. Magnified images of the cleaved caspase 3-positive microglia were also shown with an asterisk in each image. Scale bar, 50 μm. (J) Percentages of cleaved caspase 3-positive microglia quantified by double-immunofluorescence staining as shown in (I) (n = 3 each). Data were presented as mean ± SD. Unpaired t-test. ∗p *<* 0.05.

To investigate the effect of altered microglial heterogeneity on ALS pathogenesis in mice, we performed survival analyses on G93AL(B6) and G93AL(Balb) mice having the same transgene copy numbers (Figure S2A). The G93AL(Balb) mice exhibited a significantly shorter survival time than the G93AL(B6) mice, despite having comparable onset times and time of 10% body weight loss (survival time: G93AL(B6), 225.2 ± 21.7 days; G93AL(Balb), 203.2 ± 22.1 d; onset time: G93AL(B6), 145.3 ± 10.2 days; G93AL(Balb), 147.2 ± 13.2 days; time of 10% body weight loss: G93AL(B6), 201.7 ± 14.6 days; G93AL(Balb), 191.9 ± 23.9 days; disease duration: G93AL(B6), 79.9 ± 24.0 days; G93AL(Balb), 55.9 ± 18.1 d, mean ± SD) (Figures 2A−2F), indicating that G93AL(Balb) mice accelerated the late phase of disease progression. Although the sample number was very small, it was previously reported that another low-copy SOD1^G93A^ mice with the BALB/c background also had a shorter survival time than those with the C57BL/6 background.^23^

To investigate microglia and astrocyte phenotypes, we next performed triple-immunofluorescence staining with antibodies against Iba-1, Mac2, and glial fibrillary acidic protein (GFAP) in the G93AL(B6) and G93AL(Balb) spinal cords at the disease end-stage. Despite comparable microglial activation in both strains, the number of G93AL(B6) microglia exceeded that of G93AL(Balb) microglia (Figure 2G). However, astrocyte morphology, gene expression of astrocyte marker, *Gfap*, and matured oligodendrocyte numbers did not differ between these strains (Figures 2G and S2B−S2D).

Flow cytometric analysis, which was performed to clarify the time course of microglial number during disease progression by staining the spinal cord microglia with cell surface marker CD45 and to distinguish them from lymphocytes and monocytes based on a lower level of CD45 expression (Figures S3A and S3B), revealed that the G93AL(B6) and G93AL(Balb) spinal cords displayed a gradual increase and decrease in microglial number after disease onset, respectively, compared to each WT (Figure 2H).

To clarify the cause of the decreased microglial number of in the G93AL(Balb) spinal cord, we analyzed microglial apoptosis. Double-immunofluorescence staining with antibodies against cleaved caspase 3 and Iba-1, which was performed to identify apoptotic microglia, showed that the G93AL(Balb) spinal cord displayed a higher number of apoptotic microglia than the G93AL(B6) spinal cord at the disease end-stage (Figures 2I and 2J), suggesting the defective proliferation and survival of G93AL(Balb) microglia.

### Defective production of macrophage colony stimulating factor in microglia was observed in low-copy SOD1^G93A^ mice with the BALB/c background

**Figure 3:**
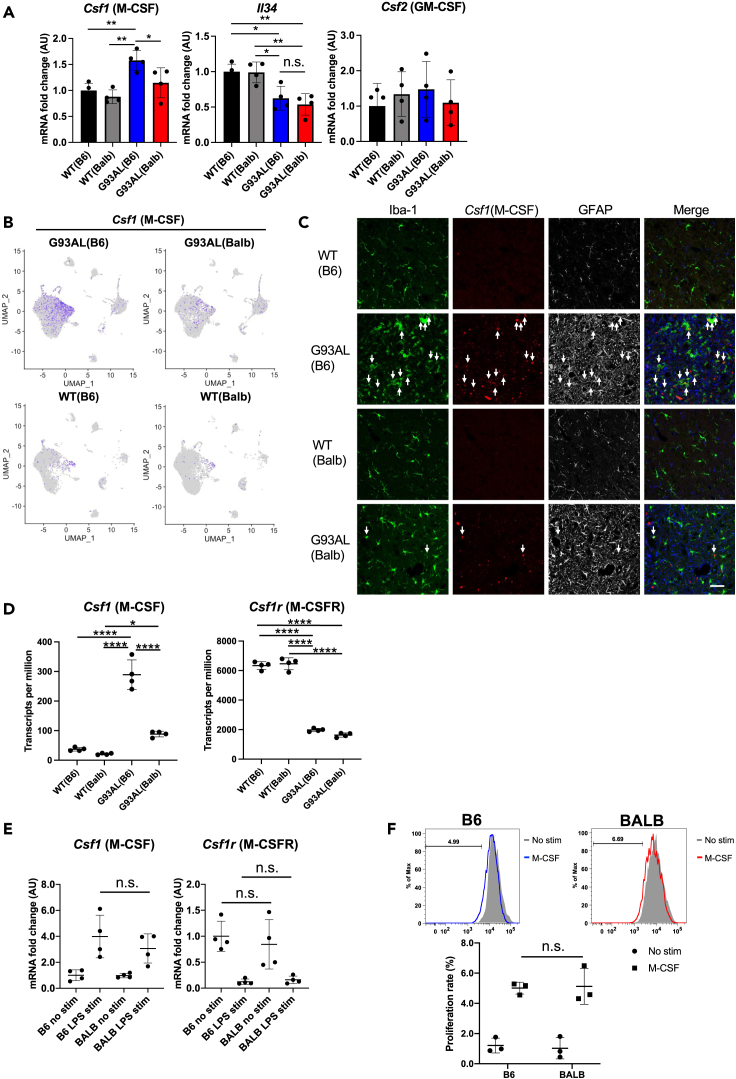
Insufficient production of M-CSF in microglia of low-copy SOD1^G93A^ mice with the BALB/c background (A) Relative mRNA levels of microglia growth factors determined by quantitative RT-PCR in the lumbar spinal cord at the disease end-stage (n = 4 each). *Csf1* (M-CSF) expression was marginally induced in G93AL(Balb) mice. Data were represented as mean ± SD. One-way ANOVA followed by Tukey–Kramer multiple comparison post hoc tests. ∗p *<* 0.05, ∗∗p < 0.01, n.s.; not significant. (B) UMAP plots showing the expression level of *Csf1* (M-CSF) and distributions of cells expressing the gene. (C) Representative images of immunofluorescence staining combined with *in situ* hybridization along with the merged images. After detecting M-CSF-expressing cells (red) by *in situ* hybridization, microglia (green) and astrocytes (white or blue) were stained with anti-Iba-1 and anti-GFAP antibodies, respectively. Arrows indicate M-CSF-producing microglia. M-CSF was mainly produced by microglia itself in G93AL(B6) mice, whereas the number of M-CSF-positive signals was lower in G93AL(Balb) mice. Scale bar, 50 μm. (D) The mRNA expression levels of *Csf1* (M-CSF) and *Csf1r* (M-CSFR) as normalized transcripts per million values in MACS-isolated microglia from spinal cords were quantified by RNA sequencing analysis (n = 4 each). Data are expressed as mean ± SD. One-way ANOVA followed by Tukey–Kramer multiple comparison post hoc tests. ∗p < 0.05, ∗∗∗∗p *<* 0.0001. (E) The mRNA expression of *Csf1* (M-CSF) and *Csf1r* (M-CSFR) in LPS-stimulated primary cultured microglia derived from WT C57BL/6 (B6) and BALB/c mice was quantified by quantitative RT-PCR. No differences were observed in *Csf1* and *Csf1r* expression (n = 4 each). Data were represented as mean ± SD. One-way ANOVA followed by Tukey–Kramer multiple comparison post hoc tests. n.s., not significant. (F) The proliferation of M-CSF-stimulated primary cultured microglia derived from WT C57BL/6 (B6) and BALB/c mice was quantified with Cytotell dye staining (n = 3 each). Distributed fluorescence dye intensities and the proportions of proliferating cells were quantified by flow cytometry. Data were represented as mean ± SD. One-way ANOVA followed by Tukey–Kramer multiple comparison post hoc tests. n.s., not significant.

To determine whether the defective production of microglia growth factors affected microglia survival and proliferation in the G93AL(Balb) spinal cord, we examined the expression of microglia growth factors, including macrophage colony stimulating factor (M-CSF), IL-34, and granulocyte-macrophage-colony-stimulating factor (GM-CSF), in the lumbar spinal cords at the disease end-stage. While M-CSF expression was strongly induced in G93AL(B6) spinal cords, its expression was hardly induced in G93AL(Balb) spinal cords (Figure 3A). Meanwhile, decreased IL-34 expression levels were observed in both ALS models compared with each WT (Figure 3A). Reduced IL-34 expression levels may reflect the loss of motor neurons in both ALS models, as IL-34 is mainly produced by neurons.^24^

The scRNA-seq analysis revealed that microglia, especially the DAM cluster mainly express M-CSF in G93AL(B6), but the number of M-CSF-expressing microglia was lower in G93AL(Balb) (Figure 3B). To confirm the results, we next identified the M-CSF-expressing cells in the spinal cords by double-immunofluorescence staining combined with *in situ* hybridization. M-CSF-expressing cells were detected by *in situ* hybridization, after which microglia and astrocyte were stained with antibodies against Iba-1 and GFAP in the spinal cords at the disease end-stage. Similar to the scRNA-seq result, M-CSF was found to be mainly produced by microglia themselves in the G93AL(B6) spinal cord, while its number was lower in the G93AL(Balb) spinal cord (Figure 3C).

To confirm our findings, we isolated microglia from the spinal cords by magnetic-activated cell sorting (MACS) and quantified mRNA levels by RNA sequencing analysis. M-CSF expression was significantly induced in G93AL(B6) microglia but marginally induced in G93AL(Balb) microglia, while the M-CSF receptor (M-CSFR) expression did not differ between these strains (Figure 3D). The decreased expression of M-CSFR in both ALS model microglia may be attributed to microglial activation. These results suggest that these phenotypes are achieved through the cell-autonomous effect caused by different genetic backgrounds.

Therefore, we compared microglial phenotypes *in vitro* using primary cultured microglia derived from C57BL/6J and BALB/c mice. However, no differences were observed in the expression levels of M-CSF and M-CSFR in LPS-stimulated microglia (Figure 3E) or in the microglial proliferation of M-CSF-stimulated microglia (Figure 3F). These results indicate that extracellular environments *in vivo* may influence ALS pathogenesis by altering microglial growth and response.

### Altered neuroinflammation- and neuroprotection-related gene expression and limited infiltration of peripheral immune cells were observed in low-copy SOD1^G93A^ mice with the BALB/c background

**Figure 4:**
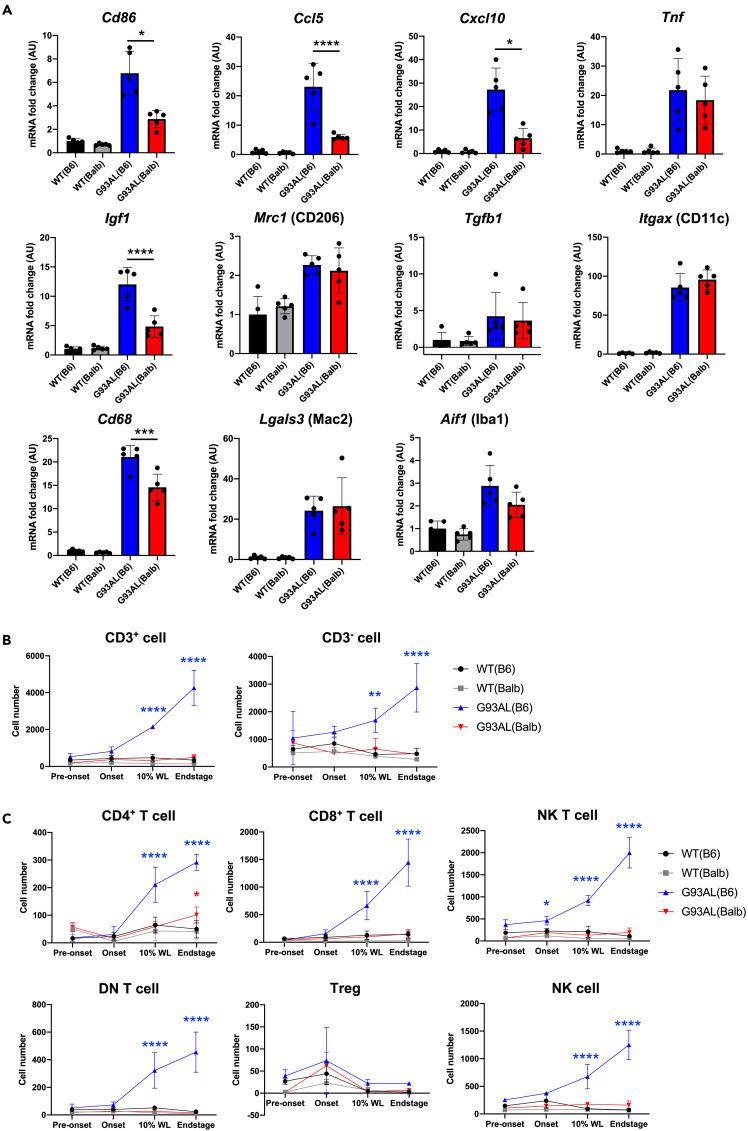
Altered neuroinflammation- and neuroprotection-related gene expression and limited infiltration of peripheral immune cells were observed in low-copy SOD1^G93A^ mice with the BALB/c background (A) Relative mRNA levels of inflammation-, microglia- or astrocyte-related molecules determined by quantitative RT-PCR in the lumbar spinal cords of G93AL mice at the disease end-stage together with age-matched WT of each strain (n = 5 each). Data were represented as mean ± SD. One-way ANOVA followed by Tukey-Kramer multiple comparison post hoc tests or Brown–Forsythe and Welch ANOVA followed by Dunnett’s T3 multiple comparison post hoc tests. ∗p *<* 0.05, ∗∗∗p *<* 0.001, ∗∗∗∗p *<* 0.0001. (B and C) The numbers of infiltrated CD45^high^/CD3^+^ cells, CD45^high^/CD3^−^ cells, and subsets of immune cells isolated from spinal cords were quantified by flow cytometric analysis at pre-onset (130 dA), onset (145 dA), 10% body weight loss (10%WL) (WT(B6) and G93AL(B6): 200 dA, WT(Balb) and G93AL(Balb): 190 dA), and end-stage of the disease (pre-onset, 10%WL, and end-stage: n = 3 each; onset: WT(B6), G93AL(B6), and WT(Balb), n = 3 each; G93AL(Balb), n = 5). Although CD4^+^ T, CD8^+^ T, NK-T, CD4^−^/CD8^−^ double-negative (DN)-T and NK cells were mainly infiltrated in G93AL(B6) spinal cords time-dependently after onset, these infiltrations were limited in G93AL(Balb) mice. Data were represented as mean ± SD. two-way ANOVA followed by Tukey–Kramer multiple comparison post hoc tests at each time point. ∗p *<* 0.05, ∗∗p *<* 0.01, ∗∗∗∗p *<* 0.0001; G93AL(B6) vs. WT(B6) as indicated by blue color; G93AL(Balb) vs. WT(Balb) as indicated by red color.

To examine the changes in neuroinflammation- and neuroprotection-related gene expressions, we quantified the expression of activation markers of microglia, proinflammatory and phagocytic markers, and a neurotrophic factor by qRT-PCR. Although the expression of the typical activation marker of microglia, *Lgals3* (Mac2), did not significantly differ between G93AL(B6) and G93AL(Balb) lumbar spinal cords, the induction of proinflammatory markers (*Cd86*, *Ccl5*, and *Cxcl10*), a phagocytotic marker (*Cd68*), and a neurotrophic factor (*Igf1*), in G93AL(Balb) lumbar spinal cords was weaker than in G93AL(B6) (Figure 4A).

A subpopulation of T-lymphocytes can infiltrate the spinal cord and exert a neuroprotective effect by inducing IGF-1 production from microglia in SOD1^G93A^ mice.^16^ As *Ccl5* and *Cxcl10* chemokines and *Igf1* were weakly expressed in the G93AL(Balb) lumbar spinal cord, we further examined the infiltration of immune cells into the spinal cord during the disease course. While the infiltration of CD45^high^/CD3^+^ cells (T-lymphocytes) and CD45^high^/CD3^−^ cells (non-T-lymphocytes) incrementally increased after onset in the G93AL(B6) spinal cords, it was scarcely observed in G93AL(Balb) spinal cords (Figure 4B). The main populations of immune cells infiltrating the G93AL(B6) spinal cord were CD8^+^ T-lymphocytes (CD8^+^-T), natural killer T-lymphocytes (NK-T), and natural killer (NK) cells (Figure 4C). These results corroborated our previous research on SOD1^G93A^ spinal cords carrying a high copy number of transgenes.^25^

RNA sequencing analysis of MACS-isolated microglia at disease end-stage revealed a distinct gene expression profile in low-copy SOD1^G93A^ microglia with the BALB/c background compared to M1, M2, or DAM microglia.

**Figure 5:**
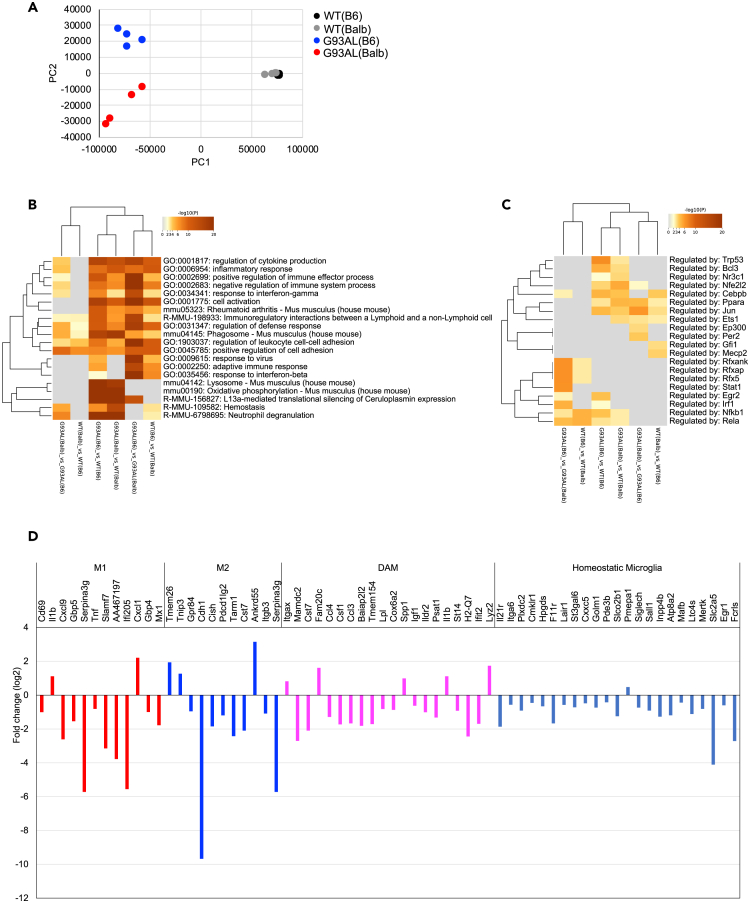
RNA sequencing analysis of MACS-isolated microglia at disease end-stage showed a distinct gene expression profile in low-copy SOD1^G93A^ microglia with the BALB/c background compared to M1, M2, or DAM microglia (A) PCA of gene expression profiles of MACS-isolated microglia at the disease end-stage of G93AL mice as well as age-matched WT mice with each strain (n = 4 each). (B and C) Gene enrichment analysis of upregulated genes among all pairs of strains by Metascape (B) or TRRUST (C). (D) Most of the M1, M2, and DAM marker genes were downregulated in G93AL(Balb) microglia relative to G93AL(B6) microglia. Relative fold changes in the indicated genes are plotted.

To elucidate the differences in microglial responses and properties between G93AL(B6) and G93AL(Balb), we directly isolated microglia from the spinal cords by MACS and performed RNA sequencing analysis at the disease end-stage. Principal-component analysis (PCA) revealed that the gene expression profiles of microglia were largely separated between G93AL(B6) and G93AL(Balb) mice (Figure 5A). Gene enrichment analysis of upregulated genes (Fold change >1.5, *q* < 0.05) among all pairs of strains revealed that some gene sets in G93AL(B6) microglia were specifically enriched in gene ontology (GO) terms of “response to interferon-gamma,” “regulation of leukocyte cell-cell adhesion,” “response to virus,” “adaptive immune response,” and “response to interferon-beta” and Reactome pathway of “immunoregulatory interactions between a lymphoid and a non-lymphoid cell” (Figure 5B). Consistent with the gene enrichment analysis, TRRUST (transcriptional regulatory relationships unraveled by sentence-based text mining)^26^ analysis also revealed that the identified gene sets were specifically regulated by transcription factors of RFX (regulatory factor binding to the X-box) complex that regulate MHC-class II (major histocompatibility complex class II) molecules and STAT1 (signal transducer and activator of transcription 1), a downstream of cytokine signaling pathways (Figure 5C).

The comparison of the expression levels of the top 30 genes^3^^,^^27^ predominantly expressed in M1, M2, DAM, and homeostatic microglia subtypes between G93AL(Balb) microglia and G93AL(B6) microglia, which was undertaken to examine phenotypic shifts, revealed that while some genes were upregulated, most of them were downregulated in G93AL(Balb) microglia (Figures 5D and S4A−S4C). It seems that most of the G93AL(Balb) microglial genes are shifted from homeostatic microglia to DAM, but the gene profile was not consistent with typical DAM (Figures S4B and S4C), suggesting that G93AL(Balb) microglia exhibit a distinct gene expression profile. In addition, gene enrichment analysis revealed that a downregulated gene set in G93AL(Balb) microglia was enriched in GO terms of “T cell activation” and “response to interferon-beta,” while an upregulated one was enriched in terms of “regulation of cell adhesion” and “actin filament-based process” (Figures S5A and S5B).

### Reclustering analysis of single-cell microglia subsets revealed that some parts of disease-associated microglia subsets slightly increased in G93AL(Balb) mice

**Figure 6:**
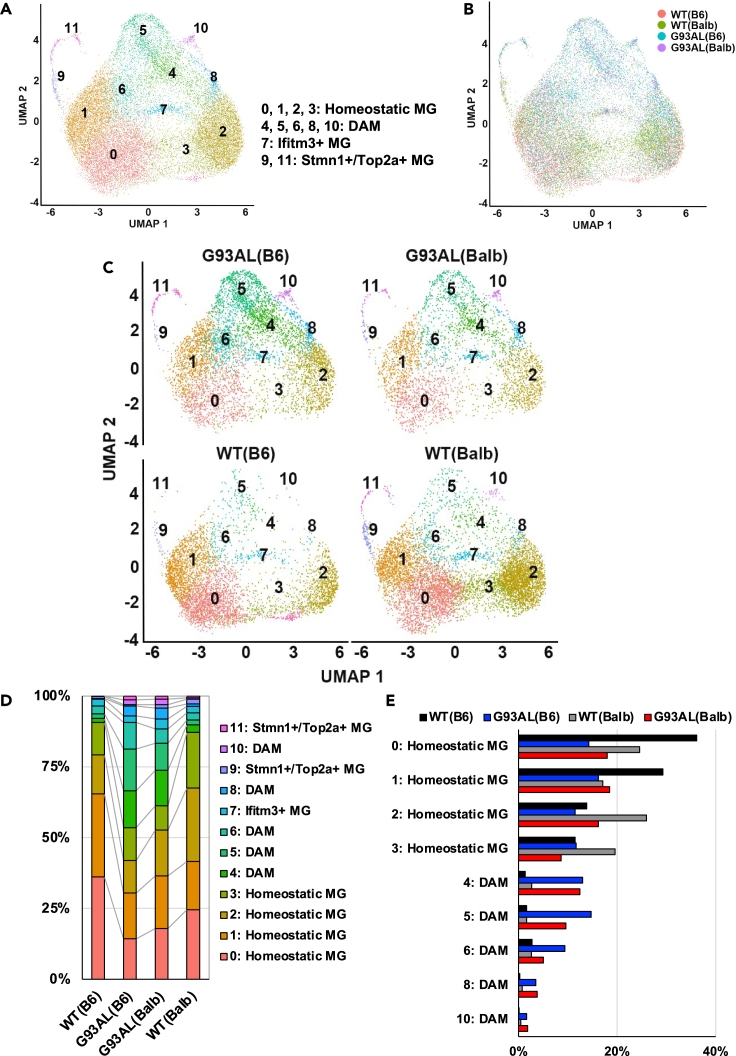
Reclustering analysis of single-cell microglia subsets showed that some parts of DAM subsets slightly increased in low-copy SOD1^G93A^ microglia with the BALB/c background (A–C) All single-cell microglia subsets (21,952 cells) of UMAP plots of WT(B6), WT(Balb), G93AL(B6), and G93AL(Balb) mice (4,742 cells from WT(B6), 7,765 cells from WT(Balb), 5,932 cells from G93AL(B6), and 3,513 cells from G93AL(Balb)). Each cluster was colored by microglia subtypes (A and C) or mouse strains (B). (D) Percentages of each microglia subtype in single-cell transcriptome plots from each strain were plotted. (E) Percentages of each homeostatic microglia cluster and each DAM cluster from each strain were plotted.

A reclustering analysis of single-cell microglia subsets, which was performed to investigate strain-specific variations in microglial diversity and their effects on disease progression, revealed 12 separate clusters of microglia subsets, including homeostatic MG (clusters 0, 1, 2, and 3), DAM (clusters 4, 5, 6, 8, and 10), Ifitm3+ MG (cluster 7), Stmn1+/Top2a+ MG (clusters 9 and 11), each with characteristic marker gene expressions (Figures 6A, 6B, S6A, and S6B; Table S1). The results were consistent with those in Figure 1, indicating that most microglia in both wild-type mice comprised homeostatic MG clusters (clusters 0, 1, 2, and 3). In addition, WT(B6) microglia predominantly consisted of clusters 0 and 1 which were highly expressing *Socs3* (Figures S6A and S6B), whereas WT(Balb) microglia were mainly represented by clusters 0, 2, and 3 (Figures 6C−6E). Furthermore, we observed that G93AL(B6) microglia were found to exhibit increased ratios of DAM clusters (4, 5, 6, 8, and 10) with decreasing ratios of homeostatic MG clusters (0 and 1), whereas G93AL(Balb) microglia displayed slightly increased ratios of the DAM clusters with decreasing ratios of homeostatic MG clusters (0, 2, and 3) (Figures 6C−6E). Notably, the ratios of DAM clusters 5 and 6 in G93AL(B6) microglia exceeded those in G93AL(Balb) microglia (Figures 6D and 6E).

### Part of disease-associated microglia cluster 5 in G93AL(B6) microglia exhibited neuroprotective properties

**Figure 7:**
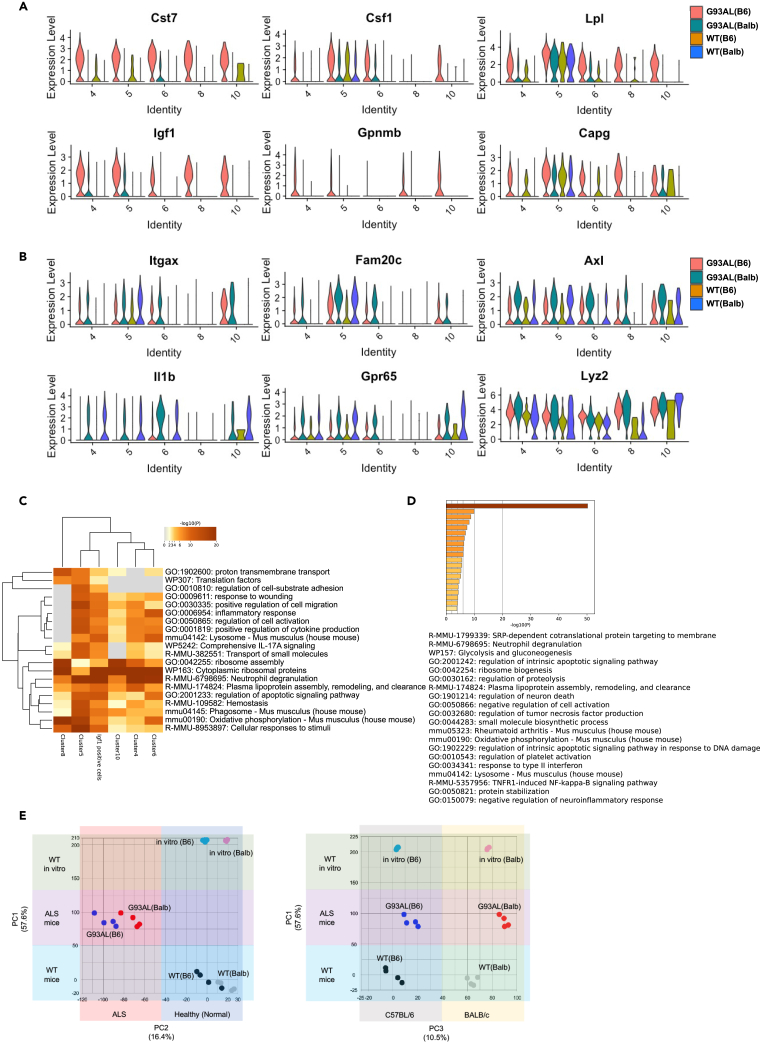
A portion of DAM cluster 5 in low-copy SOD1^G93A^ microglia with the C57BL/6 background displayed neuroprotective properties (A and B) Violin plots showing DAM marker gene expressions and distributions in each DAM cluster and genotype. The genes especially upregulated in G93AL(B6) (A) or G93A(Balb) (B) were shown. (C) Gene enrichment analysis by Metascape among DEGs of DAM clusters and *Igf1*-expressing microglia. (D) Gene enrichment analysis by Metascape in common genes between DEGs of *Igf1-*positive microglia and upregulated genes in cluster 5 of G93AL(B6) versus in that of G93AL(Balb). (E) PCA of gene expression profiles among isolated microglia from WT(B6), WT(Balb), G93AL(B6), and G93AL(Balb) spinal cords and primary cultured microglia from WT(B6) (n = 4) and WT(Balb) (n = 3) mice (*in vitro* (B6) and *in vitro* (Balb)).

The comparison of the DAM marker gene expressions of each DAM cluster among all strains undertaken to thoroughly investigate the characteristics of DAM subtypes showed that the expression levels of DAM marker genes were particularly enriched in cluster 5 (Figure S7), with some DAM marker genes being oppositely regulated between G93AL(B6) and G93AL(Balb) microglia (Figures 7A and 7B). Specifically, *Igf1* was highly expressed and distributed in G93AL(B6) DAM clusters 4, 5, 8, and 10, whereas *Csf1* was highly expressed and distributed in G93AL(B6) DAM clusters 5, 6, and 10 (Figures 7A, S7, and S8A). In addition, *Igf1*-expressing cells barely co-expressed *Csf1* (Figure S8B).

Because IGF-1 expression, especially in microglia, plays a neuroprotective role in ALS disease,^16^^,^^28^^,^^29^ we next focused on *Igf1-*expressing microglia and examined their gene expression profiles. *Igf1*-positive cells were sorted based on their expression levels (read count >2) to obtain differentially expressed genes (DEGs) (log fold-change >0.25, adjusted p < 0.05) (data not shown). DEGs from each DAM cluster (data not shown) (log fold-change >0.25, adjusted p < 0.05) were also obtained, and all the obtained DEGs were then compared using Metascape’s gene enrichment analysis. We found that DEGs of *Igf1*-positive cells mostly overlapped with those of DAM cluster 5 (Figure S8C) and were enriched in GO terms and Reactome pathways of “response to wounding,” “inflammatory response,” “positive regulation of cell migration,” “Neutrophil degranulation,” and “regulation of apoptotic signaling pathway” in common with DAM cluster 5 (Figure 7C). These results indicate that a part of *Igf1*-expressing DAM cluster 5, especially in G93AL(B6) mice, could exert a neuroprotective effect.

To extract genes that could exhibit neuroprotective functions, we merged the DEGs of *Igf1*-positive microglia with upregulated genes in cluster 5 of G93AL(B6) versus that of G93AL(Balb) (log fold-change >0.25, adjusted p < 0.05), from which one-third of each gene list was found to overlapped (Figure S8D). Similar to the Howell group report,^7^ ribosomal genes were mostly enriched in the common 153 genes as “SRP-dependent co-translational protein targeting to membrane” of the Reactome pathway, implying that some experimental conditions may have affected this result, as mentioned by them. Meanwhile, the common genes, with the exception of their ribosomal genes, were enriched in GO terms of “negative regulation of cell activation” (including *Apoe*, *Cst7*, *Cd74*, *Lgals3*, *Tnfaip3*, *Tyrobp*, *Trem2*, *Gpnmb*, *Flt1*, and *Igf1*), “regulation of neuron death” (including *Apoe*, *Csf1*, *Igf1*, *Tyrobp*, *Trem2*, and *Gpnmb*), “response to type II interferon” (including *Cd74*, *Csf1*, *Flt1*, *Lgals3*, *Mif*, and *Trem2*) and “negative regulation of neuroinflammatory response” (including *Cst7*, *Igf1*, *Trem2*, *Tnfaip3*, *Gpnmb*, *Flt1*, *Tyrobp*, *Apoe*, *Csf1*, *Cd74*, and *Lgals3*) (Figure 7D; Table S2). Notably, neuroprotective functions of IGF-1, GPNMB, Galectin-3 encoded by *Lgals3*, and Mif (macrophage migration inhibitory factor) have been reported in ALS model mice.^28^^,^^29^^,^^30^^,^^31^^,^^32^^,^^33^ CD74 can function as an Mif receptor.^34^ *Flt1* encodes VEGFR1 (vascular endothelial growth factor receptor 1), and the genetic association of VEGF with ALS and its neurotrophic function in ALS model mice have been previously reported.^35^^,^^36^ Based on our accumulating evidence, we infer that part of DAM cluster 5 in G93AL(B6) microglia exhibits neuroprotective properties.

To determine whether a cell-autonomous (intracellular) or non-cell-autonomous (extracellular) factor significantly affects microglial phenotypes, we compared the gene expression profiles of microglia isolated from all strains and primary cultured microglia from WT mice (*in vitro* (B6) and *in vitro* (Balb)). PCA revealed that the contribution ratio of PC1 likely derived from multiple environmental effects was highest (57.6%), while those of PC2 likely derived from differences between healthy (normal) and ALS disease (with mutant SOD1) and PC3 likely derived from a cell-autonomous effect caused by the clear separation of each primary cultured microglial cell were 16.4% and 10.5%, respectively (Figure 7E). Overall, our *in vitro* results (Figures 3E and 3F) and these results indicate that cell-autonomous differences in gene expression alone cannot account for all microglial phenotype variations *in vivo*. Notably, inbred C57BL/6 and BALB/c strains usually exhibit biased peripheral helper T cell responses, Th1, and Th2, respectively. At the disease end-stage, we found altered ratios of peripheral immune cell populations and Th1- or Th2- biased peripheral immune responses between G93AL(B6) and G93AL(Balb) mice (Figures S9A−S9F), indicating that the peripheral immune environment may affect disease progression by regulating microglial heterogeneity, survival, and DAM induction.

## Discussion

This study establishes for the first time that genetic background variations affect microglia heterogeneity, their responses, and disease progression in ALS model mice. The use of scRNA-seq analysis identified characteristic markers of microglia subtypes. Intriguingly, even in WT mice, a shift in genetic background from C57BL/6 to BALB/c altered the distribution of homeostatic microglia subtypes, significantly suppressing the induction of the neurotrophic factor-producing DAM subtype and the production of microglia growth factor in ALS model mice. As determined by PCA and detailed analyses of peripheral immune cells, microglia heterogeneity was found to correlate with the systemic immune environment in ALS model mice.

We found that G93AL(Balb) mice exhibited faster disease progression in the late phase than G93AL(B6) mice (Figures 2A−2F) and that the induction of DAM clusters 5 and 6 in G93AL(B6) microglia was stronger than that in G93AL(Balb) mice (Figures 6A−6D). Importantly, the expression levels of DAM marker genes were particularly enriched in cluster 5, and the DEGs of *Igf1-*positive cells mostly overlapped with those of cluster 5 (Figures 7C and S8C). The expression levels of neuroprotective genes *Igf1*, *Gpnmb*, *Lgals3*, and *Mif*, which have been previously reported in ALS model mice,^28^^,^^29^^,^^30^^,^^31^^,^^32^^,^^33^ were found to be enriched in the overlapped 153 genes (Figure S8D) as well as *Csf1* and *Tnfaip3*. We found that the induction of *Csf1*-encoded M-CSF was limited in G93AL(Balb) microglia (Figures 3A−3D). Notably, M-CSF can function as a microglial growth factor and neurotrophic factor.^37^ The anti-inflammatory activity of *Tnfaip3*-encoded A20 has been reported.^38^ Therefore, we concluded that a part of DAM cluster 5 in G93AL(B6) microglia could confer neuroprotection against ALS progression.

We also identified *Trem2*, *Apoe*, and *Tyrobp* in the overlapped 153 genes of cluster 5 and DEGs of *Igf1*-positive cells. While they were increasingly expressed in all DAM clusters of G93AL(B6), *Apoe* expression increased in those of G93AL(Balb) and *Trem2* and *Tyrobp* expression only slightly increased in them (Figure S10). Intriguingly, *Trem2* and *Tyrobp* expression levels in the homeostatic microglia clusters of WT(B6) exceeded those of WT(Balb), while *Apoe* expression was the opposite (Figure S10). Notably, *TREM2* (triggering receptor expressed on myeloid cell 2) and *APOE* (apolipoprotein E), which are major risk factors in AD, are essential to induce a DAM/MGnD/ARM subtype in both ALS and AD model mice.^3^^,^^4^^,^^5^ DAP12 encoded by *Tyrobp*, a downstream molecule of TREM2, could also contribute to DAM induction. Therefore, our results suggest that elevated expression levels of *Trem2*/*Tyrobp* and *Apoe* may be required for the induction of DAM clusters 5 and 6, while the elevated expression of *Apoe* alone may be sufficient to induce other DAM clusters. Although Amit and colleagues revealed two stages of DAM transition that depend on whether or not *Trem2* is expressed,^3^ we could not clearly detect any difference in the expression level of *Trem2* among DAM clusters, even in G93AL(B6) mice (Figure S10). However, our results corroborated those of Howell and colleagues who reported no evidence for TREM2-dependent DAM transition even in AD model mice.^7^ Overall, elevated *Trem2* expression may be necessary for inducing neuroprotective DAM clusters, although the existence of two stages of DAM transition remains unclear, indicating the need for further study to clarify TREM2 involvement in neuroprotective DAM induction in ALS. Although several genetic *TREM2* variants have been identified in patients with ALS, whether these *TREM2* variants act as ALS risk factors is being debated.^39^^,^^40^^,^^41^ Recently, the anti-inflammatory and phagocytic clearance roles of TREM2 have been identified as potential therapeutic targets for ALS.^42^^,^^43^

The scRNA-seq analysis of microglia revealed for the first time that, unlike WT(Balb) mice, the distribution of homeostatic MG clusters in WT(B6) mice was skewed toward clusters 0 and 1, which exhibited high *Socs3* expression (Figures 6C, 6D, S6A, and S6B; Table S1). SOCS3 (suppressor of cytokine signaling 3) is a protein that plays a crucial role in negatively regulating cytokine signaling pathways by binding to Janus kinase 2 (JAK2). Several cytokines including interleukin 6 (IL-6), IL-10, and interferon gamma (IFN-γ) induce this protein in macrophages and neutrophils.^44^^,^^45^ Hence, the observed bias in homeostatic microglial subpopulations in WT(B6) mice may be attributable to differences in spinal cord cytokine environments. Notably, *Il6* expression was higher in WT(B6) microglia than in WT(Balb) microglia (Figure S4A, M1 marker genes). Furthermore, because the C57BL/6 strain exhibits biased peripheral helper T cell responses toward IFN-γ-producing Th1 (Figures S9C−S9F), IFN-γ derived from peripheral CD4^+^ helper T cells may also contribute to this difference. In addition, we newly identified distinct marker genes including *Socs3* that separate each microglial cluster (Figures S6A and S6B; Table S1). Specifically, *Klf2* (Kruppel-like factor 2), *Ier2* (immediate-early response gene 2), *Socs3*, *Fosb*, and *Crybb1* expressions separate homeostatic MG subtypes into four clusters (Table S1). *Klf2*, *Ier2*, and *Fosb* are immediate-early genes (IEGs) that encode transcriptional factors immediately induced by environmental stimuli. Both Barres and Kaminska groups independently identified IEGs expressing microglial subpopulations using single-cell analyses, but the former group suggested that the sorting procedure could induce IEG expressions such as *Klf2*, *Ier2*, and *Fosb*, as these expressions were hardly detected on microglia *in vivo.*^2^^,^^46^^,^^47^ Therefore, *Socs3* and *Crybb1* should be more appropriate markers to distinguish these homeostatic MG subpopulations such as *Socs3*^high^/*Crybb1*^high^ (cluster 0), *Socs3*^high^/*Crybb1*^low^ (cluster 1), *Socs3*^−^ (cluster 2), and *Socs3*^low^/*Crybb1*^low^ (cluster 3).

Finally, we compared gene expression profiles among isolated microglia from both WT and ALS model mice of each strain and cultured microglia of each strain to assess the effect of environmental factors derived from genetic background variations on microglial phenotypes. PCA revealed that the contribution ratio derived from the cell-autonomous effect (PC3) was only 10.5% (Figure 7E). No differences in the expression levels of *Csf1* and *Csf1r* and the proliferation of cultured microglia were observed between C57BL/6 and BALB/c (Figures 3E and 3F). Therefore, we inferred that environmental factors strongly affect microglia phenotypes. Amit and colleagues compared microglia between germ-free (GF) mice and control mice at postnatal and adult ages and revealed that microglia from GF mice exhibited the deregulation of many genes associated with microglial development and immune responses,^48^ indicating that the immune system, such as intestinal immunity, strongly associates with microglial functions related to both brain development and neuroinflammation of neurodegenerative disease. Since the biased peripheral helper T cell responses between inbred C57BL/6 and BALB/c strains are traditionally known, these differences could also affect microglia development and their immune functions. Notably, Th1- or Th2-biased peripheral immune responses were observed in both WT and ALS model mice for each strain (Figures S9C–S9F). In addition, differences in the numbers of infiltrating immune cells and the ratios of peripheral immune cells were observed between G93AL(B6) and G93AL(Balb) mice at the disease end-stage (Figures 4B, 4C, S9A, and S9B). However, our previous research revealed that the depletion of CD8^+^ T, natural killer T (NK-T), and natural killer (NK) cells in ALS model mice with the C57BL/6 genetic background from the onset of age had no impact on survival times,^25^ indicating that environmental factors derived from peripheral immune cells and/or infiltrating immune cells, with the exception of CD8^+^ T, NK-T, and NK cells, may affect the course of the disease by regulating microglial heterogeneity, survival, and DAM induction in ALS model mice. Intriguingly, some DAM marker genes were found to be oppositely regulated between G93AL(B6) and G93AL(Balb) microglia (Figures 7A and 7B). Further analyses will be required to reveal whether these differences could be linked to the disease mechanisms of ALS.

In summary, our findings provide new evidence that genetic diversity may affect microglia heterogeneity, their responses, and disease progression in an ALS model. Identifying the therapeutic targets of ALS will require further study to elucidate the details of environmental factors regulating microglial heterogeneity and their neuroprotective functions.

### Limitations of the study

This study suggests that genetic background variation affects microglial heterogeneity, their responses, and disease progression in ALS model mice. However, since this study conducted the experiments only between two strains, further analysis among ALS model mice with various genetic backgrounds will be required. In addition, although the comparative analyses of gene expression between *in vivo* and *in vitro* microglia suggest the contribution of immune system-derived environmental factors on microglial heterogeneity and functions, further validation using ALS model mice crossed with immunoregulatory gene knockout or transgenic mice will also be required.

## STAR★Methods

### Key resources table

**Table:** 

REAGENT or RESOURCE	SOURCE	IDENTIFIER
**Antibodies**		

Rabbit polyclonal anti-GFAP	DAKO	Cat# Z0334, RRID:AB_10013382
Mouse monoclonal anti-GFAP	Sigma-Aldrich	Cat# G3893, RRID:AB_477010
Rat monoclonal anti-Mac2	CEDERLANE	Cat# CL8942AP, RRID:AB_10060357
Rabbit polyclonal anti-Iba1	FUJIFILM Wako	Cat#019-19741, RRID:AB_839504
Goat polyclonal anti-Iba-1	Novus	Cat# NB100-1028, RRID:AB_521594
Rabbit polyclonal anti-Cleaved Caspase 3 (Asp175)	Cell Signaling Technology	Cat# 9661, RRID:AB_2341188
Mouse monoclonal anti-APC (Ab-7)	Merck Millipore	Cat# OP80, RRID:AB_2057371
Goat anti-rabbit IgG(H+L) highly cross-absorbed secondary antibody, Alexa Fluor 488	Thermo Fisher Scientific	Cat# A11034, RRID:AB_2576217
Goat anti-mouse IgG(H+L) highly cross-absorbed secondary antibody, Alexa Fluor 488	Thermo Fisher Scientific	Cat# A11029, RRID:AB_2534088
Goat anti-mouse IgG(H+L) highly cross-absorbed secondary antibody, Alexa Fluor 633	Thermo Fisher Scientific	Cat# A21052, RRID:AB_2535719
Goat anti-rat IgG(H+L) highly cross-absorbed secondary antibody, Alexa Fluor 546	Thermo Fisher Scientific	Cat# A11081, RRID:AB_2534125
Donkey anti-goat IgG(H+L) cross-absorbed secondary antibody, Alexa Fluor 546	Thermo Fisher Scientific	Cat# A11056, RRID:AB_2534103
Sheep anti-Digoxigenin-AP, Fab fragments	Roche	Cat# 11093274910, RRID:AB_514497
Rat monoclonal anti-CD16/32	Thermo Fisher Scientific	Cat# 14-0161-82, RRID:AB_467133
Rat monoclonal anti-CD45, APC-Cy7 conjugated	BD Biosciences	Cat# 557659, RRID:AB_396774
Rat monoclonal anti-CD4, PE-Cy7 conjugated	BD Biosciences	Cat# 552775, RRID:AB_394461
Armenian Hamster monoclonal anti-CD3e, PerCP-Cy5.5 conjugated	BD Biosciences	Cat# 551163, RRID:AB_394082
Rat monoclonal anti-CD8a, FITC conjugated	BD Biosciences	Cat# 553030, RRID:AB_394568
Mouse monoclonal anti-NK1.1, PE conjugated	BD Biosciences	Cat# 553165, RRID:AB_394677
Rat monoclonal anti-CD49b, PE conjugated	BD Biosciences	Cat# 553858, RRID:AB_395094
Rat monoclonal anti-IFN-gamma, Alexa Fluor 488 conjugated	BD Biosciences	Cat# 557724, RRID:AB_396832
Rat monoclonal anti-IL-17A, Alexa Fluor 647 conjugated	BD Biosciences	Cat# 560184, RRID:AB_1645204
Rat monoclonal anti-IL-4, PE conjugated	BioLegend	Cat# 504104, RRID:AB_315317

**Chemicals, peptides, and recombinant proteins**		

0.25% Trypsin	FUJIFILM Wako	Cat# 201-18841
DNase I	Roche	Cat# 10104159001
PBS	Nissui	Cat# 05913
Poly-L-lysine solution	Sigma-Aldrich	Cat# P4707
DMEM	Gibco	Cat# C11965500
L-glutamine	FUJIFILM Wako	Cat# 073-05391
Penicillin-Streptomycin Solution (x100)	FUJIFILM Wako	Cat# 168-23191
LPS	Sigma-Aldrich	Cat# L2880
Recombinant Murine M-CSF	PEPROTECH	Cat# 315-02
Paraformaldehyde	Agar scientific	Cat# AGR1018
Tissue-Tek O.C.T compound	SAKURA finetek	Cat# 4583
Triton-X-100	Sigma-Aldrich	Cat# X100
Normal goat serum	Cederlane	Cat# CL1200
DAPI solution(1mg/mL)	Nacalai	Cat# 19178-91
Fluoromount/Plus mounting medium	Diagnostic BioSystems	Cat# K048
Diethyl pyrocarbonate	Nacalai	Cat# 12311-44
Proteinase K Solution (20 mg/mL), RNA grade	Thermo Fisher Scientific	Cat# 25530049
Deionized formamide	Thermo Fisher Scientific	Cat# AM9342
N-Lauroylsarcosine, neat	Sigma-Aldrich	Cat# L5000
Polyoxyethylene(20) Sorbitan Monolaurate (Tween 20)	FUJIFILM Wako	Cat# 9005-64-5
HBSS	Thermo Fisher Scientific	Cat# 14025092
Collagenase, Type 4	Worthington	Cat# LS004188
Percoll	Cytiva	Cat# 17089101
Ficoll-Paque PREMIUM 1.084	Cytiva	Cat# 17544602
Phorbol 12-myristate 13-acetate (PMA)	Sigma-Aldrich	Cat# P1585
Ionomycin calcium salt from Streptomyces conglobatus	Sigma-Aldrich	Cat# I0634
RPMI1640 Medium	Thermo Fisher Scientific	Cat# 11875093

**Critical commercial assays**		

DIG RNA Labeling Kit (SP6/T7)	Roche	Cat# 11175025910
Blocking Reagent For nucleic acid hybridization and detection	Roche	Cat# 11096176001
Fast Red Tablets	Roche	Cat# 11496549001 (Discontinued)
Neural Tissue Dissociation Kit−Postnatal Neurons	Miltenyi Biotec	Cat# 130-094-802
Myelin Removal Beads II, human, mouse, rat	Miltenyi Biotec	Cat# 130-096-433
CD11b (Microglia) MicroBeads, human and mouse	Miltenyi Biotec	Cat# 130-093-634
mirVana miRNA Isolation Kit, with phenol	Thermo Fisher Scientific	Cat# AM1560
RNeasy Micro Kit	QIAGEN	Cat# 74004
RNeasy Mini Kit	QIAGEN	Cat# 74104
PrimeScript RT reagent Kit (Perfect Real Time)	Takara Bio	Cat# RR037
SYBR(TB Green) Premix Ex Taq II (Tli RNaseH Plus)	Takara Bio	Cat# RR820
BD GolgiPlug Protein Transport Inhibitor (Containing Brefeldin A)	BD Biosciences	Cat# 555029, RRID:AB_2869014
BD Cytofix/Cytoperm Fixation/Permeabilization Kit	BD Biosciences	Cat# 554722, RRID:AB_2869010
CytoTell Red 650 fluorescent dye	AAT Bioquest	Cat# 22255
RNA 6000 Pico Kit	Agilent Technologies	Cat# 5067-1513
TruSeq RNA Library Prep Kit v2	illumina	Cat# RS-122-2001, RS-122-2002
MGIEasy RNA Directional Library Prep Kit V2.0	MGI Tech Co	Cat# 1000006386
BD Pharmingen 7-AAD	BD Biosciences	Cat# 559925, RRID:AB_2869266
Chromium Next GEM Single Cell 3’ Reagent Kits v3.1 for dual index	10x genomics	Cat# 1000269
Chromium Next GEM Chip G Single Cell Kit	10x genomics	Cat# 1000127
Dual Index Kit TT Set A	10x genomics	Cat# 1000215

**Deposited data**		

Raw files of RNA-seq analysis in isolated microglia	This study	NCBI-GEO:GSE252050
Raw files of RNA-seq analysis in primary cultured microglia	This study	NCBI-SRA:PRJNA1061369
Raw files of single-cell RNA-seq analysis	This study	NCBI-SRA:PRJNA1060899

**Experimental models: Organisms/strains**		

G93AL(B6)	This study	N/A
G93AL(Balb)	This study	N/A

**Oligonucleotides**		

qRT-PCR primers, see Table S3		N/A

**Software and algorithms**		

GraphPad Prism	GraphPad Software	RRID:SCR_002798
Flowjo	Flowjo, LLC	RRID:SCR_008520
R software	R Foundation	RRID:SCR_001905
RStudio	Posit Software, PBC	RRID:SCR_000432
Subio Platform	Subio inc	N/A
Trim Galore	Babraham Bioinformatics	RRID:SCR_011847
FastQC	Babraham Bioinformatics	RRID:SCR_014583
HISAT2	The Center for Computational Biology at Hohns Hopkins University	RRID:SCR_015530
BEDTools	Quinlan Laboratory at the University of Virginia	RRID:SCR_006646
StringTie	The Center for Computational Biology at Hohns Hopkins University	RRID:SCR_016323
edgeR	Bioconductor	RRID:SCR_012802
Cell Ranger	10x genomics	RRID:SCR_017344
Seurat	Satija Lab and Collaborators	RRID:SCR_016341

**Other**		

pGEM-7Zf(-) Vector	Promega	Cat# P2371

### Resource availability

#### Lead contact

Further information and requests for resources and reagents should be directed to and will be fulfilled by the lead contact, Koji Yamanaka (koji.yamanaka@riem.nagoya-u.ac.jp).

#### Materials availability

Plasmid and mouse lines generated in this study will be available from the lead contact upon request.

#### Data and code availability

• RNA-seq and single-cell RNA-seq data have been deposited at NCBI GEO and NCBI SRA and are publicly available as of the date of publication. Accession numbers are listed in the key resources table.
• All data reported in this paper will be shared by the lead contact upon request.
• This paper does not report original code.
• Any additional information required to reanalyze the data reported in this paper is available from the lead contact upon request.

### Experimental model and study participant details

#### Mice

We used SOD1^G93A^ mice carrying a low copy number of mutant SOD1^G93A^ in the C57BL/6J background (G93AL(B6)), which were spontaneously lost transgene copies (∼ −40%) during breeding of an original SOD1^G93A^ mouse (B6.Cg-Tg (SOD1∗G93A)1Gur/J) obtained from Jackson Laboratory (Strain#:004435, RRID:IMSR_JAX:004435). BALB/c congenic SOD1^G93A^ mice (G93AL(Balb)) were generated by backcrossing G93AL(B6) with BALB/cA female mice over 10 times. Comparable transgene copy numbers in each mouse were confirmed by quantitative real-time PCR (qPCR). The genotyping method of mice was previously described elsewhere.^25^ qPCR was performed by using human SOD1-f primer (CAATGTGACTGCTGACAAAG), human SOD1-r primer (GTGCGGCCAATGATGCAAT), mouse β-actin-f primer (TTGGCCTCACTGTCCACCTT), and mouse β-actin-r primer (CGGACTCATCGTACTCCTGCTT). PCR was performed by using mouse SOD-A primer (GTTACATATAGGGGTTTACTTCATAATCTG), human/mouse SOD-C primer (CAGCAGTCACATTGCCCARGTCTCCAACATG), and human SOD-B primer (CCAAGATGCTTAACTCTTGTAATCAATGGC). Mice were maintained under the standard specific pathogen free environment (12 h light-dark cycle; 23 ± 1 ºC; 50 ± 5% humidity) and were treated in accordance with the guidelines established by the Institutional Animal Care and Use Committee of Nagoya University. The experiments using genetically modified animals and organisms were approved by the Animal Care and Use Committee and the recombinant DNA experiment committee of Nagoya University. We used the same numbers of male and female mice as possible for each experiment except for the scRNA-seq analysis. scRNA-seq analysis was performed with only female mice of each strain.

#### Primary microglia culture

Brains from male and female mice at postnatal days 1-2 were dissociated in 0.25% Trypsin and 10 mg/mL DNase I containing PBS at 37 °C for 10 min. Dissociated cells were washed and plated on poly-L-Lysine coated flasks in 10% FBS DMEM (DMEM medium supplemented with 10% FBS, L-glutamine, 50 U/mL penicillin, and 50 μg /mL streptomycin) in the 5% CO_2_ incubator. After 1-2 weeks, non-adherent microglial cells were collected by shaking flasks for 2 h at 150 rpm in the 5% CO_2_ incubator. Microglia were seeded on culture dishes and stimulated with 1 μg /mL lipopolysaccharide (LPS) for 6 h or 50 ng/mL macrophage colony-stimulating factor (M-CSF) for 72 h.

### Method details

#### Survival experiments of the mice

The times of disease onset and 10% weight loss were retrospectively determined as the times when mice reached a maximum body weight and 10% weight loss from the maximum body weight, respectively, and that of end stage was determined as the time when the mouse could not right itself within 20 seconds after being placed on its side. Statistical analyses of survival time were performed with a log-rank test and an unpaired t-test by using GraphPad Prism.

#### Immunofluorescence staining of spinal cord sections

Mice were deeply anesthetized, transcardially perfused with 4% paraformaldehyde in phosphate buffer (4% PFA), and post-fixed with 4% PFA overnight. Following post-fixation, the lumbar spinal cords dissected from the perfused mice were cryoprotected in 30% sucrose in PBS for 24 h, then embedded in Tissue-Tek O. C. T compound, and frozen. Twelve-micron cryosections were pre-incubated with 0.5 % Triton-X-100 in PBS for 30 min, and then incubated with 5 % normal goat serum / 0.3% Triton-X-100 in PBS for blocking for 1 h, followed by immunostaining with combinations of antibodies against GFAP (1:1000, rabbit polyclonal antibody or 1:500, mouse monoclonal antibody), Mac-2 (1:500), Iba1 (1:500, rabbit polyclonal antibody or 1:250, goat polyclonal antibody), cleaved caspase 3 (1:200), or APC (cc-1) (1:200) at 4°C overnight. The sections were incubated with fluorescent-dye-conjugated secondary antibodies against rabbit, rat, goat, or mouse IgGs and 2.5 μg/mL DAPI at room temperature (RT) for 2 h, followed by mounting with a drop of Fluoromount/Plus mounting medium. Imaging data were acquired using a confocal laser microscope (LSM700, Carl-Zeiss, Germany). Statistical analyses were performed using GraphPad Prism.

#### *In situ* hybridization of spinal cord sections

*In situ* hybridization was performed on 12 μm cryosections of lumbar spinal cords using a digoxigenin-labeled *Csf1*-specific cRNA probe. The cRNA probe was synthesized from pGEM-7Zf(-) vector inserted *Csf1* cDNA by using DIG RNA Labeling Kit (SP6 / T7) according to the manufacturer’s instruction. The sections were fixed with 4% PFA at RT for 15 min, washed with Diethyl pyrocarbonate (DEPC)-treated PBS, incubated with 1 μg/mL RNase-free Proteinase K Solution at 37 °C for 15 min, and then post-fixed with 4% PFA at RT for 15 min. After washing with DEPC-treated PBS, the sections were incubated with hybridization buffer (50% deionized formamide / 2% Blocking Reagent / 0.1% N-Lauroylsarcosine / 0.1% SDS / 5× SSC (pH 4.5)) containing the cRNA probe at 70 °C overnight in a slide mailer. Following hybridization with the cRNA probe, the sections were washed with 50% deionized formamide / 1% SDS / 2× SSC (pH 4.5) solution at 70 °C for 30 min, three times, washed with 0.1 M TrisHCl (pH 7.5) / 150 mM NaCl / 0.05% Tween 20 solution at RT for 5 min, twice, and then incubated with 0.5% Blocking Reagent / 0.1 M TrisHCl (pH 7.5) / 150 mM NaCl / 0.05% Tween 20 solution for blocking at RT for 30 min. After blocking, the sections were incubated with an alkaline phosphatase-conjugated anti-digoxigenin antibody (1:1000) at RT for 2 h , washed with 0.1 M TrisHCl (pH 7.5) / 150 mM NaCl / 0.05% Tween 20 solution at RT for 10 min, twice, and then stained with Fast Red substrate according to the manufacturer’s instruction. Immunofluorescence staining was conducted after *in situ* hybridization procedure.

#### Isolation of microglia from spinal cords by magnetic-activated cell sorting

Spinal cord microglia were isolated by using magnetic-activated cell sorting (MACS) technique. Deeply anesthetized mice were transcardially perfused with PBS to remove blood from blood vessels. The spinal cords were then dissected and dissociated in Neural Tissue Dissociation Kit-Postnatal Neurons reagents by using gentleMACS Dissociator (Miltenyi Biotec, Germany) at 37 °C for 15 min. For removal of myelin debris, the cells were incubated with Myelin Removal Beads II according to the manufacturer’s instruction and purified by autoMACS Pro Separator (Miltenyi Biotec, Germany). The cells were then treated with anti-CD16/CD32 antibody (2 μg/mL) for blocking Fc receptors, followed by reacting with CD11b (Microglia) MicroBeads according to the manufacturer’s instruction. CD11b-positive microglia were isolated by autoMACS Pro Separator (Miltenyi Biotec, Germany).

#### Quantitative PCR

Total RNAs from lumbar spinal cords, MACS-isolated microglia, or cultured microglia were purified using mirVana miRNA Isolation Kit or RNeasy Micro Kit according to the manufacturer’s instructions. cDNAs were synthesized using PrimeScript RT reagent Kit (Perfect Real Time) and were subjected to quantitative PCR with the following protocol: 1 cycle at 95 °C for 30 s, 40 cycles at 95 °C for 5 s and 60 °C for 30 s, with SYBR (TB Green) Premix Ex Taq II (Tli RNaseH Plus) by using the Thermal Cycler Dice Real Time System II (Takara Bio, Japan). The value of each gene was calculated in duplicate and averaged. The list of gene-specific primer pairs was shown in Table S3. Statistical analyses were performed using GraphPad Prism.

#### Flow cytometric analysis

Spinal cords, dissected from mice transcardially perfused with PBS for blood removal, were minced into 1mm^3^ pieces in collagenase working solution (1 mg/mL Collagenase Type 4 / 0.4 mg/mL DNase I in HBSS) and incubated at 37 °C for 15 min. For removal of myelin debris, the cells were resuspended in 37% Percoll in PBS, and then centrifuged at 780 × g for 20 min. After the removal of myelin debris in the upper layer, a cell pellet containing microglia and immune cells was collected. Splenocytes were collected by mashing the spleen with slide glasses, followed by lysing red blood cells with an ammonium chloride solution. For the flow cytometry analysis, Fc receptors were blocked with 2 μg/mL anti-CD16/CD32 antibody on ice for 10 min, and then the cells were stained with combinations of the following antibodies, anti-CD45-APC-Cy7, anti-CD4-PE-Cy7, anti-CD3e-PerCP-Cy5.5, anti-CD8a-FITC, anti-NK1.1-PE, or anti-CD49b-PE at RT for 20 min. Data were obtained by using FACS Verse flow cytometer (BD Biosciences, USA) and further analyzed using Flowjo Software. Representative gating strategies for microglia and immune cells were shown in Figures S3A and S3B. Statistical analyses were performed using GraphPad Prism.

#### Intracellular cytokine staining

Peripheral bloods were collected from the central tail artery, and then peripheral blood mononuclear cells (PBMCs) were isolated by centrifugation (400 × g for 30 min) with Ficoll-Paque PREMIUM 1.084. Splenocytes were obtained by the method mentioned above. Immune cells were stimulated with 10 ng/mL Phorbol 12-myristate 13-acetate (PMA) and 1μg/mL Ionomycin in the presence of BD GolgiPlug protein transport inhibitor (1:1000) in 10% FBS containing RPMI1640 medium for 4hrs at 37 °C / 5% CO_2_, and then cell surface staining was performed with anti-CD45-APC-Cy7, anti-CD4-PE-Cy7, anti-CD8a-FITC, and anti-CD3e-PerCP-Cy5.5 by the method mentioned above. After cell surface staining, the cells were fixed and permeabilized with BD Cytofix/Cytoperm solution, and then intracellular cytokine staining was performed with anti-IFN-γ-alexa fluor 488, anti-IL-17A-alexa fluor 647, and anti-IL-4-PE according to the manufacturer’s instruction. Flow Cytometry analyses were performed by using FACS Verse flow cytometer (BD Biosciences, USA). The ratio of cytokine producing cells in the CD4-positive T-lymphocytes were analyzed by using Flowjo Software. Statistical analyses were performed using GraphPad Prism.

#### Proliferation analysis of microglia

Primary cultured microglia were seeded on culture dishes. On the next day, microglia were stained with CytoTell Red 650 fluorescent dye at 37 °C for 30 min in the 5% CO_2_ incubator, and then washed with PBS. Microglia labeled with CytoTell Red 650 were stimulated with 50 ng/mL macrophage colony-stimulating factor (M-CSF) in 10 % FBS DMEM medium for 72 h. The fluorescence changes in divided microglia were then monitored using FACS Verse flow cytometer (BD Biosciences, USA). The ratio of divided microglia was analyzed using Flowjo Software. Statistical analyses were performed using GraphPad Prism.

#### RNA sequencing

Total RNAs from MACS-isolated microglia and primary cultured microglia were purified using RNeasy Micro Kit (QIAGEN, Germany) and RNeasy Mini Kit (QIAGEN, Germany) according to the manufacturer’s instructions, respectively. The RNA integrity was analyzed by 2100 Bioanalyzer (Agilent Technologies, USA) with RNA 6000 pico Kit. Libraries were prepared with TruSeq RNA Library Prep Kit v2 (Illumina, USA) or MGIEasy RNA Directional Library Prep Kit V2.0 (MGI Tech Co, China) and sequenced with 151-nt paired-end reads using HiSeq X (Illumina, USA) or DNBSEQ-G400 (MGI Tech Co, China).

#### Analysis of RNA sequencing data

The mouse mm10 reference genome assembly and gene annotation from the UCSC genome browser were retrieved from iGenomes (https://support.illumina.com/sequencing/sequencing_software/igenome.html). Removal of adapter sequences and filtering of low-quality bases (quality score < 20) were performed with the Trim Galore v0.5.0 (https://www.bioinformatics.babraham.ac.uk/projects/trim_galore/). Reads were qualified with FastQC v0.11.8 (https://www.bioinformatics.babraham.ac.uk/projects/fastqc/) before and after trimming. The processed reads were mapped on the mouse mm10 genome assembly by using HISAT2 v2.1.0^49^ with read strand specificity information, followed by removal of alignments on rDNA regions with BEDTools v2.25.0.^50^ Gene expressions were quantified from the mapping data with StringTie v1.3.5.^51^ TPM (Transcripts Per Million) and CPM (Counts Per Million) values for the individual genes were computed with the StringTie and edgeR v.3.24.3^52^ implemented in R v.3.5.1, respectively. Differential expression analysis between two groups with replicates was performed with edgeR, and the *q*-value was calculated for multiple testing correction of the *p*-values with the *q*-value package v2.24.1^53^ in R. Principal Component Analysis (PCA) for the samples was performed with the prcomp function in R by using the CPM values or with the Subio Platform software ver. 1. 24. 5853 (Subio Inc., Amami city, Kagoshima, Japan) by using normalized read-count values. Heatmaps were drawn in R with heatmap. 2 by using the TPM values.

#### Analysis of single-cell RNA sequencing data

A spinal cord, dissected from each female mouse transcardially perfused with PBS, was dissociated in Neural Tissue Dissociation Kit-Postnatal Neurons reagents by using gentleMACS Dissociator (Miltenyi Biotec, Germany) at 37°C for 15 min. To remove myelin debris, the cells were incubated with Myelin Removal Beads II (Miltenyi Biotec, Germany) according to the manufacturer’s instruction and then purified by autoMACS Pro Separator (Miltenyi Biotec, Germany). The living single-cells were sorted by FACSMelody Cell Sorter (BD Biosciences, USA) after 7-AAD (7-Aminoactinomycin D) staining. The barcoded libraries were generated by Chromium controller (10x genomics, USA) using Chromium Next GEM Single Cell 3’ Reagent Kits v3.1 for dual index and sequenced with 150-nt paired-end reads by using Illumina Hiseq 2500 (Illumina, USA). The sequencing data were aligned and processed using Cell Ranger pipelines and analyzed using the Seurat package V4.^54^ After filtering low-quality or dying cells, the data from remained cells (WT(B6): 6473 cells, WT(Balb): 9762 cells, G93AL(B6): 8391 cells, G93AL(Balb): 6590 cells) were normalized and integrated, and then a clustering analysis was performed. Identified microglia subsets (WT(B6): 4742 cells, WT(Balb): 7765 cells, G93AL(B6): 5932 cells, G93AL(Balb): 3513 cells) were also re-clustered in the same way. Differentially expressed genes (log fold-change > 0.25, adjusted *p* < 0.05) in each Disease-associated microglia cluster and *Igf1*-positive microglia were obtained with “FindAllMarkers” and “FindMarkers” functions of the Seurat, respectively.

#### Gene enrichment analysis

Gene enrichment analyses were performed on significantly deregulated gene sets using Metascape.^55^ TRRUST (transcriptional regulatory relationships unraveled by sentence-based text mining)^26^ analysis was simultaneously implemented by Metascape.

### Quantification and statistical analysis

Statistical analyses were performed with GraphPad Prism except for RNA-seq and scRNA-seq analyses. Differential expression analysis of RNA-seq between two groups with replicates was performed with edgeR, and the *q*-value was calculated for multiple testing correction of the *p*-values with the *q*-value package v2.24.1 in R. Regarding scRNA-seq analysis, differentially expressed genes (log fold-change > 0.25, adjusted *p* < 0.05) in each Disease-associated microglia cluster and *Igf1*-positive microglia were obtained with “FindAllMarkers” and “FindMarkers” functions of the Seurat, respectively. All of the statistical details of experiments can be found in the figure legends, figures, and results, including the statistical tests used, exact value of n, and what n represents. All error bars indicate the mean ± SD. The *p*-values, adjusted *p*-values, and *q*-values lower than 0.05 were considered significant. The *p*-values lower than 0.05 were represented by the following symbols in the figures: ∗*p*<0.05, ∗∗*p*<0.01, ∗∗∗*p*<0.001, ∗∗∗∗*p*<0.0001.

## References

[1] Hammond T.R., Dufort C., Dissing-Olesen L., Giera S., Young A., Wysoker A., Walker A.J., Gergits F., Segel M., Nemesh J. (2019). Single-Cell RNA Sequencing of Microglia throughout the Mouse Lifespan and in the Injured Brain Reveals Complex Cell-State Changes. Immunity.

[2] Li Q., Cheng Z., Zhou L., Darmanis S., Neff N.F., Okamoto J., Gulati G., Bennett M.L., Sun L.O., Clarke L.E. (2019). Developmental Heterogeneity of Microglia and Brain Myeloid Cells Revealed by Deep Single-Cell RNA Sequencing. Neuron.

[3] Keren-Shaul H., Spinrad A., Weiner A., Matcovitch-Natan O., Dvir-Szternfeld R., Ulland T.K., David E., Baruch K., Lara-Astaiso D., Toth B. (2017). A Unique Microglia Type Associated with Restricting Development of Alzheimer's Disease. Cell.

[4] Krasemann S., Madore C., Cialic R., Baufeld C., Calcagno N., El Fatimy R., Beckers L., O'Loughlin E., Xu Y., Fanek Z. (2017). The TREM2-APOE Pathway Drives the Transcriptional Phenotype of Dysfunctional Microglia in Neurodegenerative Diseases. Immunity.

[5] Sala Frigerio C., Wolfs L., Fattorelli N., Thrupp N., Voytyuk I., Schmidt I., Mancuso R., Chen W.T., Woodbury M.E., Srivastava G. (2019). The Major Risk Factors for Alzheimer's Disease: Age, Sex, and Genes Modulate the Microglia Response to Abeta Plaques. Cell Rep..

[6] Sobue A., Komine O., Hara Y., Endo F., Mizoguchi H., Watanabe S., Murayama S., Saito T., Saido T.C., Sahara N. (2021). Microglial gene signature reveals loss of homeostatic microglia associated with neurodegeneration of Alzheimer's disease. Acta Neuropathol. Commun..

[7] Yang H.S., Onos K.D., Choi K., Keezer K.J., Skelly D.A., Carter G.W., Howell G.R. (2021). Natural genetic variation determines microglia heterogeneity in wild-derived mouse models of Alzheimer's disease. Cell Rep..

[8] Heiman-Patterson T.D., Deitch J.S., Blankenhorn E.P., Erwin K.L., Perreault M.J., Alexander B.K., Byers N., Toman I., Alexander G.M. (2005). Background and gender effects on survival in the TgN(SOD1-G93A)1Gur mouse model of ALS. J. Neurol. Sci..

[9] Heiman-Patterson T.D., Sher R.B., Blankenhorn E.A., Alexander G., Deitch J.S., Kunst C.B., Maragakis N., Cox G. (2011). Effect of genetic background on phenotype variability in transgenic mouse models of amyotrophic lateral sclerosis: a window of opportunity in the search for genetic modifiers. Amyotroph Lateral Scler..

[10] Mancuso R., Oliván S., Mancera P., Pastén-Zamorano A., Manzano R., Casas C., Osta R., Navarro X. (2012). Effect of genetic background on onset and disease progression in the SOD1-G93A model of amyotrophic lateral sclerosis. Amyotroph Lateral Scler..

[11] Nardo G., Iennaco R., Fusi N., Heath P.R., Marino M., Trolese M.C., Ferraiuolo L., Lawrence N., Shaw P.J., Bendotti C. (2013). Transcriptomic indices of fast and slow disease progression in two mouse models of amyotrophic lateral sclerosis. Brain.

[12] Pfohl S.R., Halicek M.T., Mitchell C.S. (2015). Characterization of the Contribution of Genetic Background and Gender to Disease Progression in the SOD1 G93A Mouse Model of Amyotrophic Lateral Sclerosis: A Meta-Analysis. J. Neuromuscul. Dis..

[13] Sher R.B., Heiman-Patterson T.D., Blankenhorn E.A., Jiang J., Alexander G., Deitch J.S., Cox G.A. (2014). A major QTL on mouse chromosome 17 resulting in lifespan variability in SOD1-G93A transgenic mouse models of amyotrophic lateral sclerosis. Amyotroph. Lateral Scler. Frontotemporal Degener..

[14] Valbuena G.N., Cantoni L., Tortarolo M., Bendotti C., Keun H.C. (2019). Spinal Cord Metabolic Signatures in Models of Fast- and Slow-Progressing SOD1(G93A) Amyotrophic Lateral Sclerosis. Front. Neurosci..

[15] Clarke B.E., Patani R. (2020). The microglial component of amyotrophic lateral sclerosis. Brain.

[16] Chiu I.M., Chen A., Zheng Y., Kosaras B., Tsiftsoglou S.A., Vartanian T.K., Brown R.H., Carroll M.C. (2008). T lymphocytes potentiate endogenous neuroprotective inflammation in a mouse model of ALS. Proc. Natl. Acad. Sci. USA.

[17] Chiu I.M., Morimoto E.T.A., Goodarzi H., Liao J.T., O'Keeffe S., Phatnani H.P., Muratet M., Carroll M.C., Levy S., Tavazoie S. (2013). A neurodegeneration-specific gene-expression signature of acutely isolated microglia from an amyotrophic lateral sclerosis mouse model. Cell Rep..

[18] Rangaraju S., Dammer E.B., Raza S.A., Rathakrishnan P., Xiao H., Gao T., Duong D.M., Pennington M.W., Lah J.J., Seyfried N.T., Levey A.I. (2018). Identification and therapeutic modulation of a pro-inflammatory subset of disease-associated-microglia in Alzheimer's disease. Mol. Neurodegener..

[19] McCombe P.A., Lee J.D., Woodruff T.M., Henderson R.D. (2020). The Peripheral Immune System and Amyotrophic Lateral Sclerosis. Front. Neurol..

[20] Kang S.H., Li Y., Fukaya M., Lorenzini I., Cleveland D.W., Ostrow L.W., Rothstein J.D., Bergles D.E. (2013). Degeneration and impaired regeneration of gray matter oligodendrocytes in amyotrophic lateral sclerosis. Nat. Neurosci..

[21] Kang S.H., Fukaya M., Yang J.K., Rothstein J.D., Bergles D.E. (2010). NG2+ CNS glial progenitors remain committed to the oligodendrocyte lineage in postnatal life and following neurodegeneration. Neuron.

[22] Magnus T., Carmen J., Deleon J., Xue H., Pardo A.C., Lepore A.C., Mattson M.P., Rao M.S., Maragakis N.J. (2008). Adult glial precursor proliferation in mutant SOD1G93A mice. Glia.

[23] Acevedo-Arozena A., Kalmar B., Essa S., Ricketts T., Joyce P., Kent R., Rowe C., Parker A., Gray A., Hafezparast M. (2011). A comprehensive assessment of the SOD1G93A low-copy transgenic mouse, which models human amyotrophic lateral sclerosis. Dis. Model. Mech..

[24] Greter M., Lelios I., Pelczar P., Hoeffel G., Price J., Leboeuf M., Kündig T.M., Frei K., Ginhoux F., Merad M., Becher B. (2012). Stroma-derived interleukin-34 controls the development and maintenance of langerhans cells and the maintenance of microglia. Immunity.

[25] Komine O., Yamashita H., Fujimori-Tonou N., Koike M., Jin S., Moriwaki Y., Endo F., Watanabe S., Uematsu S., Akira S. (2018). Innate immune adaptor TRIF deficiency accelerates disease progression of ALS mice with accumulation of aberrantly activated astrocytes. Cell Death Differ..

[26] Han H., Cho J.W., Lee S., Yun A., Kim H., Bae D., Yang S., Kim C.Y., Lee M., Kim E. (2018). TRRUST v2: an expanded reference database of human and mouse transcriptional regulatory interactions. Nucleic Acids Res..

[27] Satoh J.-i. (2018). Gene expression profiles of M1 and M2 microglia characterized by comparative analysis of public datasets. Clin. Exp. Neuroimmunol..

[28] Dodge J.C., Haidet A.M., Yang W., Passini M.A., Hester M., Clarke J., Roskelley E.M., Treleaven C.M., Rizo L., Martin H. (2008). Delivery of AAV-IGF-1 to the CNS extends survival in ALS mice through modification of aberrant glial cell activity. Mol. Ther..

[29] Kaspar B.K., Lladó J., Sherkat N., Rothstein J.D., Gage F.H. (2003). Retrograde viral delivery of IGF-1 prolongs survival in a mouse ALS model. Science.

[30] Tanaka H., Shimazawa M., Kimura M., Takata M., Tsuruma K., Yamada M., Takahashi H., Hozumi I., Niwa J.i., Iguchi Y. (2012). The potential of GPNMB as novel neuroprotective factor in amyotrophic lateral sclerosis. Sci. Rep..

[31] Lerman B.J., Hoffman E.P., Sutherland M.L., Bouri K., Hsu D.K., Liu F.T., Rothstein J.D., Knoblach S.M. (2012). Deletion of galectin-3 exacerbates microglial activation and accelerates disease progression and demise in a SOD1(G93A) mouse model of amyotrophic lateral sclerosis. Brain Behav..

[32] Leyton-Jaimes M.F., Benaim C., Abu-Hamad S., Kahn J., Guetta A., Bucala R., Israelson A. (2016). Endogenous macrophage migration inhibitory factor reduces the accumulation and toxicity of misfolded SOD1 in a mouse model of ALS. Proc. Natl. Acad. Sci. USA.

[33] Leyton-Jaimes M.F., Kahn J., Israelson A. (2019). AAV2/9-mediated overexpression of MIF inhibits SOD1 misfolding, delays disease onset, and extends survival in mouse models of ALS. Proc. Natl. Acad. Sci. USA.

[34] Leng L., Metz C.N., Fang Y., Xu J., Donnelly S., Baugh J., Delohery T., Chen Y., Mitchell R.A., Bucala R. (2003). MIF signal transduction initiated by binding to CD74. J. Exp. Med..

[35] Lambrechts D., Storkebaum E., Morimoto M., Del-Favero J., Desmet F., Marklund S.L., Wyns S., Thijs V., Andersson J., van Marion I. (2003). VEGF is a modifier of amyotrophic lateral sclerosis in mice and humans and protects motoneurons against ischemic death. Nat. Genet..

[36] Wang Y., Mao X.O., Xie L., Banwait S., Marti H.H., Greenberg D.A., Jin K. (2007). Vascular endothelial growth factor overexpression delays neurodegeneration and prolongs survival in amyotrophic lateral sclerosis mice. J. Neurosci..

[37] Michaelson M.D., Bieri P.L., Mehler M.F., Xu H., Arezzo J.C., Pollard J.W., Kessler J.A. (1996). CSF-1 deficiency in mice results in abnormal brain development. Development.

[38] Voet S., Mc Guire C., Hagemeyer N., Martens A., Schroeder A., Wieghofer P., Daems C., Staszewski O., Vande Walle L., Jordao M.J.C. (2018). A20 critically controls microglia activation and inhibits inflammasome-dependent neuroinflammation. Nat. Commun..

[39] Cady J., Koval E.D., Benitez B.A., Zaidman C., Jockel-Balsarotti J., Allred P., Baloh R.H., Ravits J., Simpson E., Appel S.H. (2014). TREM2 Variant p.R47H as a Risk Factor for Sporadic Amyotrophic Lateral Sclerosis. JAMA Neurol..

[40] Rikos D., Siokas V., Mentis A.F.A., Aloizou A.M., Liampas I., Tsouris Z., Peristeri E., Stamati P., Hadjigeorgiou G.M., Dardiotis E. (2022). TREM2 R47H variant and risk for Alzheimer's disease: assessment in a Greek population and updated meta-analysis. Int. J. Neurosci..

[41] Siokas V., Aloizou A.M., Liampas I., Tsouris Z., Mentis A.F.A., Nasios G., Papadimitriou D., Bogdanos D.P., Hadjigeorgiou G.M., Dardiotis E. (2021). Lack of association between TREM2 rs75932628 variant and amyotrophic lateral sclerosis. Mol. Biol. Rep..

[42] Xie M., Liu Y.U., Zhao S., Zhang L., Bosco D.B., Pang Y.P., Zhong J., Sheth U., Martens Y.A., Zhao N. (2022). TREM2 interacts with TDP-43 and mediates microglial neuroprotection against TDP-43-related neurodegeneration. Nat. Neurosci..

[43] Xie M., Zhao S., Bosco D.B., Nguyen A., Wu L.J. (2022). Microglial TREM2 in amyotrophic lateral sclerosis. Dev. Neurobiol..

[44] Bode J.G., Nimmesgern A., Schmitz J., Schaper F., Schmitt M., Frisch W., Häussinger D., Heinrich P.C., Graeve L. (1999). LPS and TNFalpha induce SOCS3 mRNA and inhibit IL-6-induced activation of STAT3 in macrophages. FEBS Lett..

[45] Cassatella M.A., Gasperini S., Bovolenta C., Calzetti F., Vollebregt M., Scapini P., Marchi M., Suzuki R., Suzuki A., Yoshimura A. (1999). Interleukin-10 (IL-10) selectively enhances CIS3/SOCS3 mRNA expression in human neutrophils: evidence for an IL-10-induced pathway that is independent of STAT protein activation. Blood.

[46] Ochocka N., Kaminska B. (2021). Microglia Diversity in Healthy and Diseased Brain: Insights from Single-Cell Omics. Int. J. Mol. Sci..

[47] Ochocka N., Segit P., Walentynowicz K.A., Wojnicki K., Cyranowski S., Swatler J., Mieczkowski J., Kaminska B. (2021). Single-cell RNA sequencing reveals functional heterogeneity of glioma-associated brain macrophages. Nat. Commun..

[48] Matcovitch-Natan O., Winter D.R., Giladi A., Vargas Aguilar S., Spinrad A., Sarrazin S., Ben-Yehuda H., David E., Zelada González F., Perrin P. (2016). Microglia development follows a stepwise program to regulate brain homeostasis. Science.

[49] Kim D., Paggi J.M., Park C., Bennett C., Salzberg S.L. (2019). Graph-based genome alignment and genotyping with HISAT2 and HISAT-genotype. Nat. Biotechnol..

[50] Quinlan A.R., Hall I.M. (2010). BEDTools: a flexible suite of utilities for comparing genomic features. Bioinformatics.

[51] Pertea M., Pertea G.M., Antonescu C.M., Chang T.C., Mendell J.T., Salzberg S.L. (2015). StringTie enables improved reconstruction of a transcriptome from RNA-seq reads. Nat. Biotechnol..

[52] Robinson M.D., McCarthy D.J., Smyth G.K. (2010). edgeR: a Bioconductor package for differential expression analysis of digital gene expression data. Bioinformatics.

[53] Storey J.D., Tibshirani R. (2003). Statistical significance for genomewide studies. Proc. Natl. Acad. Sci. USA.

[54] Hao Y., Hao S., Andersen-Nissen E., Mauck W.M., Zheng S., Butler A., Lee M.J., Wilk A.J., Darby C., Zager M. (2021). Integrated analysis of multimodal single-cell data. Cell.

[55] Zhou Y., Zhou B., Pache L., Chang M., Khodabakhshi A.H., Tanaseichuk O., Benner C., Chanda S.K. (2019). Metascape provides a biologist-oriented resource for the analysis of systems-level datasets. Nat. Commun..

