# Supplementary material for: Genetic background variation impacts microglial heterogeneity and disease progression in amyotrophic lateral sclerosis model mice
Source: iScience. 2024 Jan 11;27(2):108872. doi: 10.1016/j.isci.2024.108872 (PMC10839647; doi:10.1016/j.isci.2024.108872)
Supplement: Document S1. Figures S1–S10 and Tables S1–S3 [file mmc1.pdf]

iScience, Volume *27*

Supplemental information

Genetic background variation impacts microglial heterogeneity and disease progression in amyotrophic lateral sclerosis model mice

Okiru Komine, Syuhei Ohnuma, Kunihiko Hinohara, Yuichiro Hara, Mayuko Shimada, Tomohiro Akashi, Seiji Watanabe, Akira Sobue, Noe Kawade, Tomoo Ogi, and Koji Yamanaka

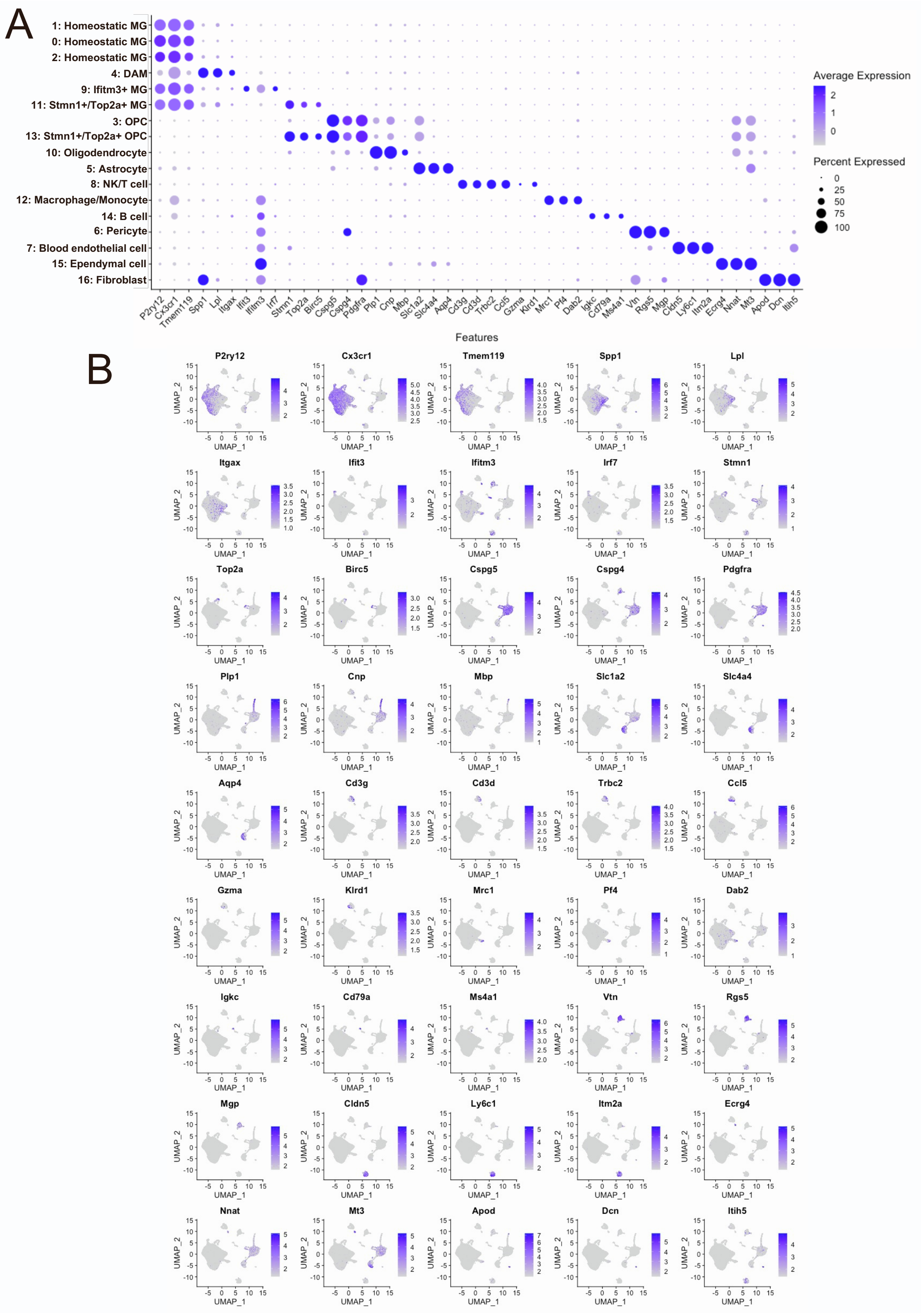
A
1: Homeostatic MG
0: Homeostatic MG
2: Homeostatic MG
4: DAM
9: Ifitm3+ MG
11: Stmn1+/Top2a+ MG
3: OPC
13: Stmn1+/Top2a+ OPC
10: Oligodendrocyte
5: Astrocyte
8: NK/T cell
12: Macrophage/Monocyte
14: B cell
6: Pericyte
7: Blood endothelial cell
15: Ependymal cell
16: Fibroblast
P2ry12 Cx3cr1 Tmem119 Spp1 Lpl Itgax Ifit3 Ifitm3 Irf7 Stmn1 Top2a Birc5 Cspg5 Cspg4 Pdgfra Plp1 Cnp Mbp Slc1a2 Slc4a4 Aqp4 Cd3g Cd3d Trbc2 Ccl5 Gzma Klrd1 Mrc1 Pf4 Dab2 Igkc Cd79a Ms4a1 Vtn Rgs5 Mgp Cldn5 Ly6c1 Itm2a Ecrg4 Nnat Mt3 Apod Dcn Itih5
Features
Average Expression
2
1
0
Percent Expressed
0
25
50
75
100
B
P2ry12 Cx3cr1 Tmem119 Spp1 Lpl
Itgax Ifit3 Ifitm3 Irf7 Stmn1
Top2a Birc5 Cspg5 Cspg4 Pdgfra
Plp1 Cnp Mbp Slc1a2 Slc4a4
Aqp4 Cd3g Cd3d Trbc2 Ccl5
Gzma Klrd1 Mrc1 Pf4 Dab2
Igkc Cd79a Ms4a1 Vtn Rgs5
Mgp Cldn5 Ly6c1 Itm2a Ecrg4
Nnat Mt3 Apod Dcn Itih5
UMAP_1
UMAP_2

Figure S1. Characterization of each cluster in 31,216 spinal cord cells from all strains, Related to Figure 1

(A) Dot plot showing averaged expression levels (color intensity) of cell-type marker genes and ratios of cells expressing their genes (dot size) in each cluster. (B) UMAP plots showing the expression levels of cell-type marker genes and distributions of cells expressing their genes.

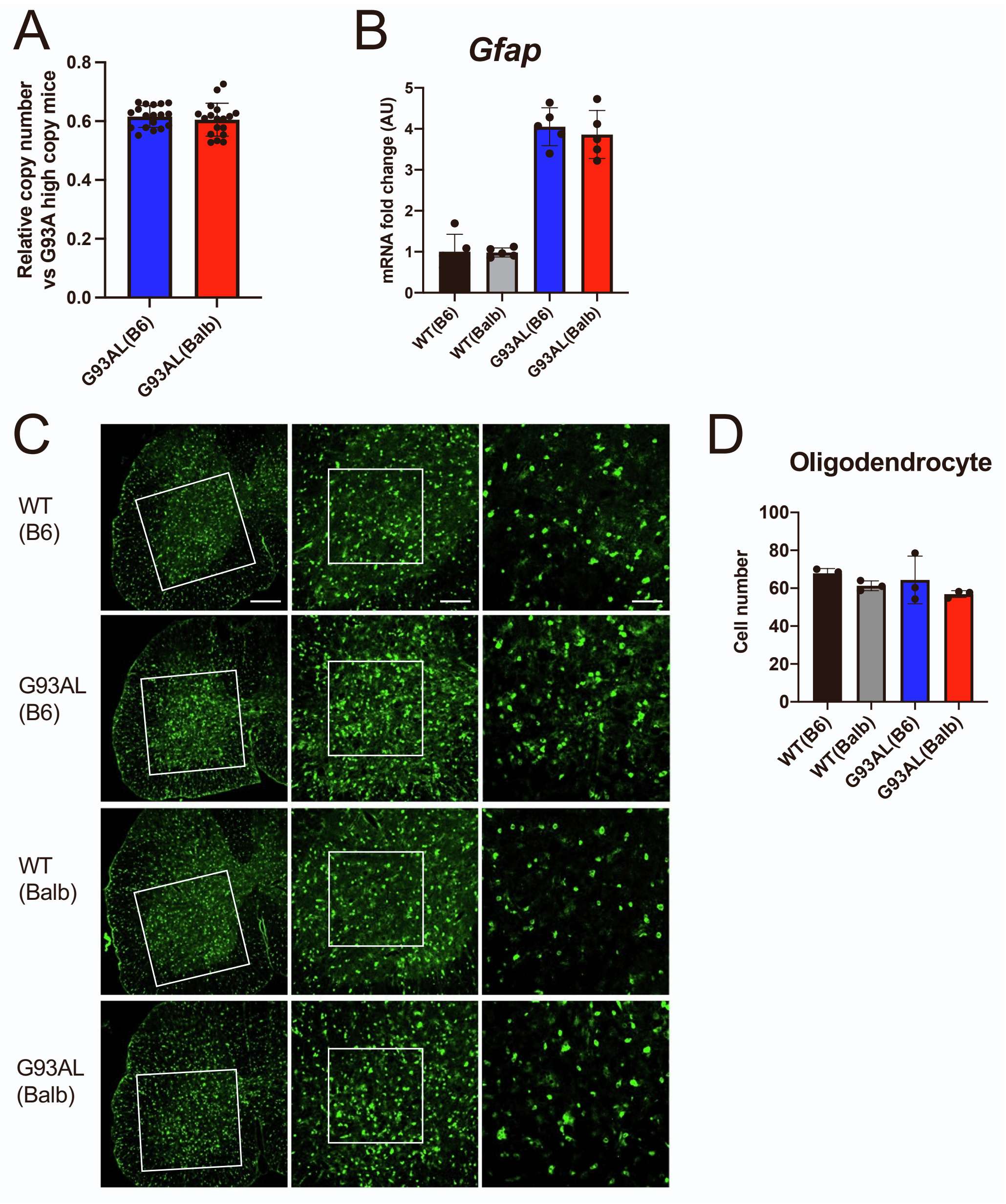

Figure S2. Numbers of astrocytes and oligodendrocytes did not differ between ALS model mice with different genetic backgrounds, Related to Figure 2

(A) The transgene copy numbers of each mouse were quantified by comparing with the original SOD1^{G93A} high copy mice (n=18, each). Data were represented as mean ± SD. Unpaired *t*-test. (B) Relative mRNA levels of *Gfap* determined by quantitative RT-PCR in the lumbar spinal cord at the disease end-stage (n = 5, each). Data were represented as mean ± SD. One-way ANOVA followed by

Tukey-Kramer multiple comparison post hoc test. (C) Representative immunofluorescence images of the lumbar spinal cord sections of G93AL(B6) and G93AL(Balb) at the disease end-stage and age-matched WT(B6) and WT(Balb) stained for APC (green). Different rates of magnification images were shown in left panels (Scale bar, 200 μm), middle panels (boxed images in left panels, Scale bar, 100 μm), and right panels (boxed images in middle panels, Scale bar, 50 μm). (D) No differences were observed in the numbers of oligodendrocytes among the genotypes (n = 3, each). Data were represented as mean ± SD. One-way ANOVA followed by Tukey–Kramer multiple comparison post hoc test.

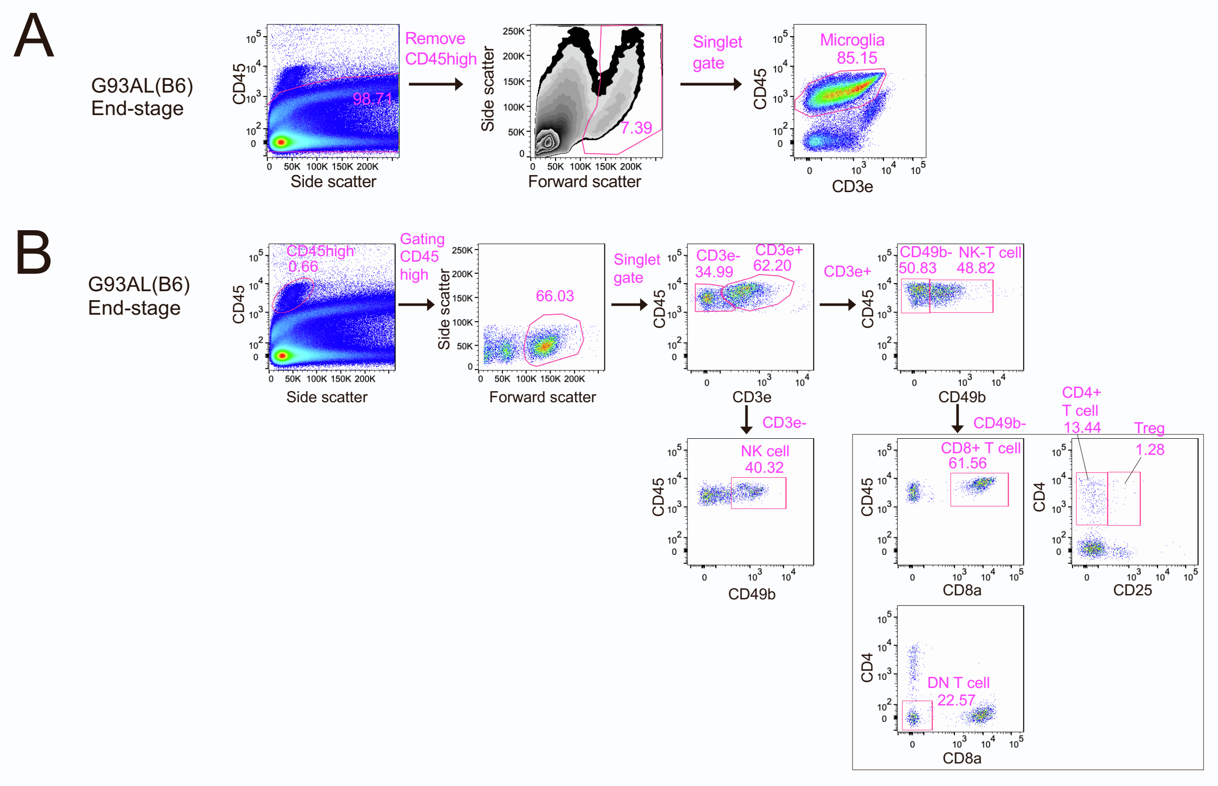

Figure S3. Representative gating strategies for microglia and immune cells, Related to Figure 2, Figure 3, and Figure S9

(A) Representative gating strategies for microglia. (B) Representative gating strategies for immune cells.

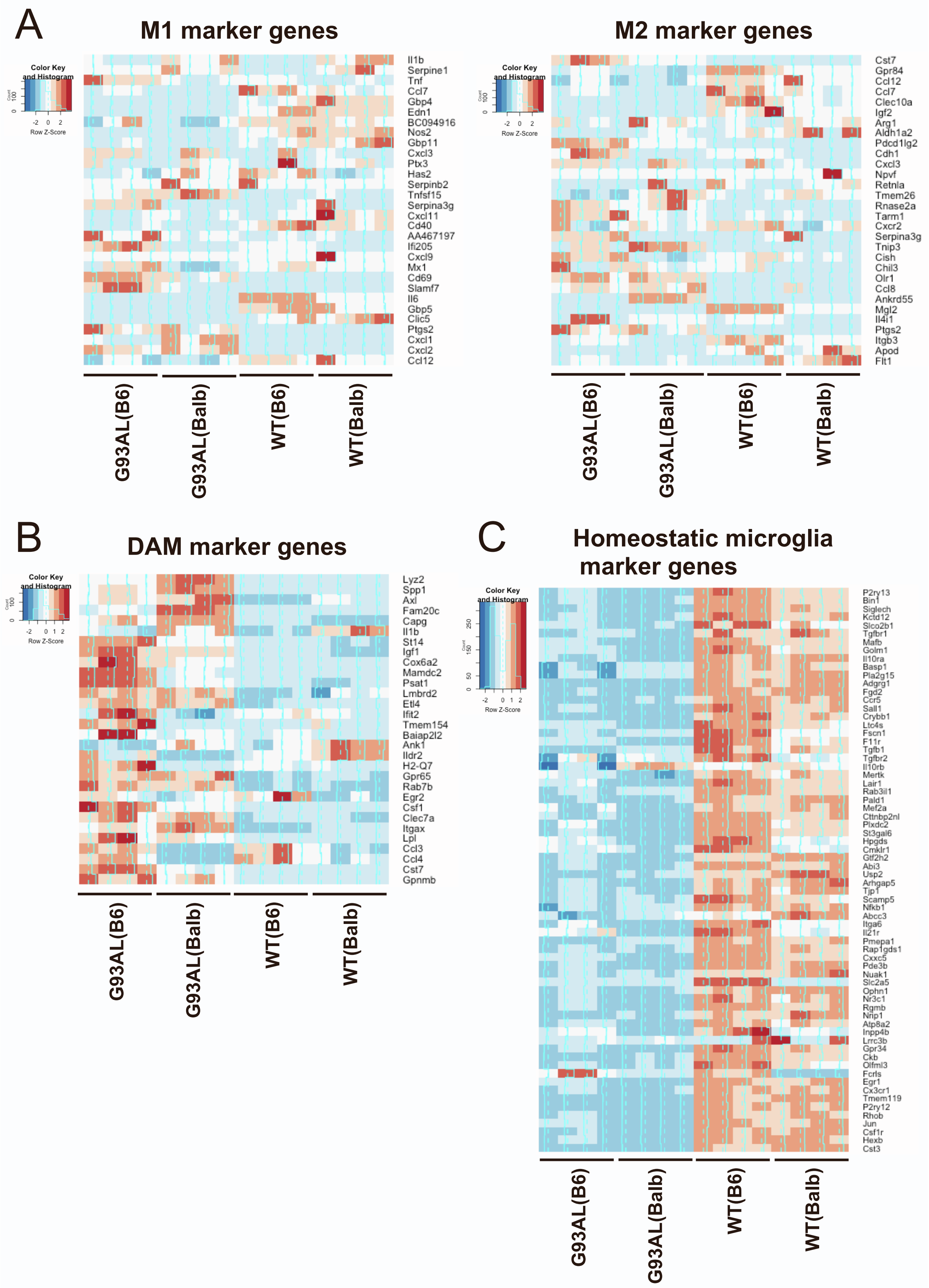
A
M1 marker genes
M2 marker genes
B
DAM marker genes
C
Homeostatic microglia marker genes
Color Key and Histogram
Row Z-Score
G93AL(B6)
G93AL(Balb)
WT(B6)
WT(Balb)
Il1b
Serpine1
Tnf
Ccl7
Gbp4
Edn1
BC094916
Nos2
Gbp11
Cxcl3
Ptx3
Has2
Serpinb2
Tnfsf15
Serpina3g
Cxcl11
Cd40
AA467197
Ifi205
Cxcl9
Mx1
Cd69
Slamf7
Il6
Gbp5
Clic5
Ptgs2
Cxcl1
Cxcl2
Ccl12
Cst7
Gpr84
Ccl12
Ccl7
Clec10a
Igf2
Arg1
Aldh1a2
Pdcd1lg2
Cdh1
Cxcl3
Npvf
Retnla
Tmem26
Rnase2a
Tarm1
Cxcr2
Serpina3g
Tnip3
Cish
Chil3
Olr1
Ccl8
Ankrd55
Mgl2
Il4i1
Ptgs2
Itgb3
Apod
Flt1
Lyz2
Spp1
Axl
Fam20c
Capg
Il1b
St14
Igf1
Cox6a2
Mamdc2
Psat1
Lmbrd2
Etl4
Ifit2
Tmem154
Baiap2l2
Ank1
Ildr2
H2-Q7
Gpr65
Rab7b
Egr2
Csf1
Clec7a
Itgax
Lpl
Ccl3
Ccl4
Cst7
Gpnmb
P2ry13
Bin1
Siglech
Kctd12
Slco2b1
Tgfbr1
Mafb
Golm1
Il10ra
Basp1
Pla2g15
Adgrg1
Fgd2
Ccr5
Sall1
Crybb1
Ltc4s
Fscn1
F11r
Tgfb1
Tgfbr2
Il10rb
Mertk
Lair1
Rab3il1
Pald1
Mef2a
Cttnbp2nl
Plxdc2
St3gal6
Hpgds
Cmklr1
Gtf2h2
Abi3
Usp2
Arhgap5
Tjp1
Scamp5
Nfkb1
Abcc3
Itga6
Il21r
Pmepa1
Rap1gds1
Cxxc5
Pde3b
Nuak1
Slc2a5
Ophn1
Nr3c1
Rgmb
Nrip1
Atp8a2
Inpp4b
Lrrc3b
Gpr34
Ckb
Olfml3
Fcrls
Egr1
Cx3cr1
Tmem119
P2ry12
Rhob
Jun
Csf1r
Hexb
Cst3

Figure S4. Heatmaps showing expression levels of the known microglia subtype marker genes in isolated microglia from each genotype, Related to Figure 5

(A, B, and C) Heatmaps showing expression levels of M1 and M2 microglia marker genes (A), DAM marker genes (B), and homeostatic microglia marker genes (C) (n = 4, each).

A

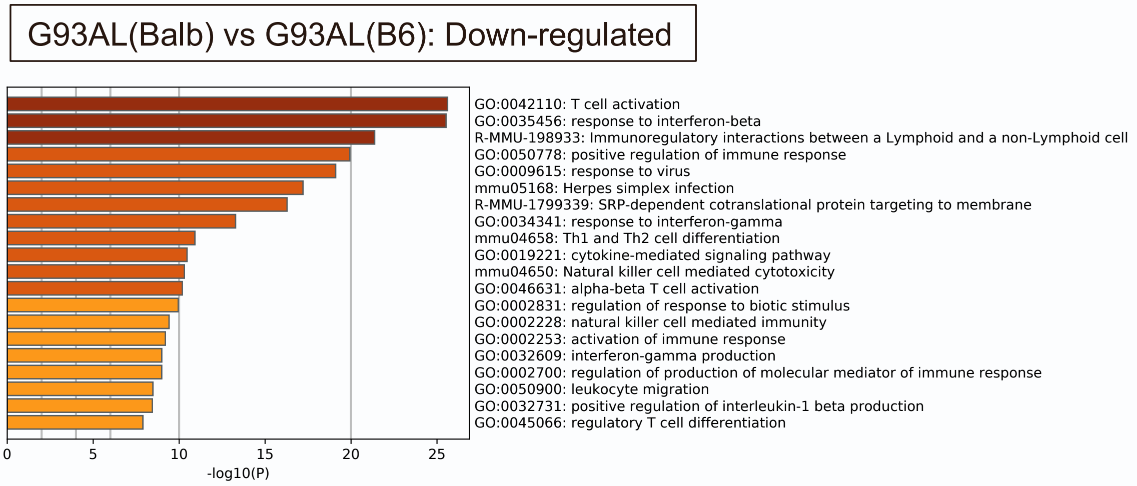

B

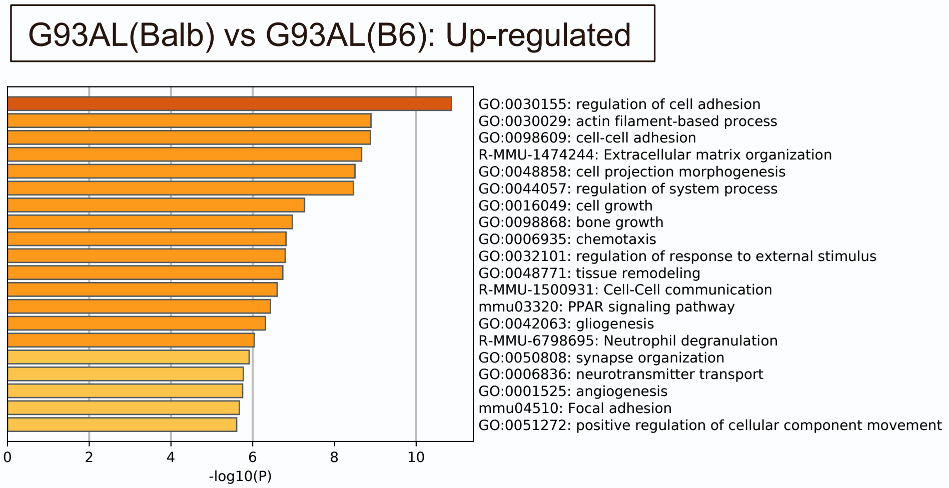

Figure S5. Gene enrichment analyses of upregulated and downregulated genes between isolated ALS model microglia with different genetic backgrounds, Related to Figure 5

(A and B) Gene enrichment analyses by Metascape in downregulated (A) and upregulated (B) genes between isolated G93AL(Balb) microglia versus G93AL(B6) microglia.

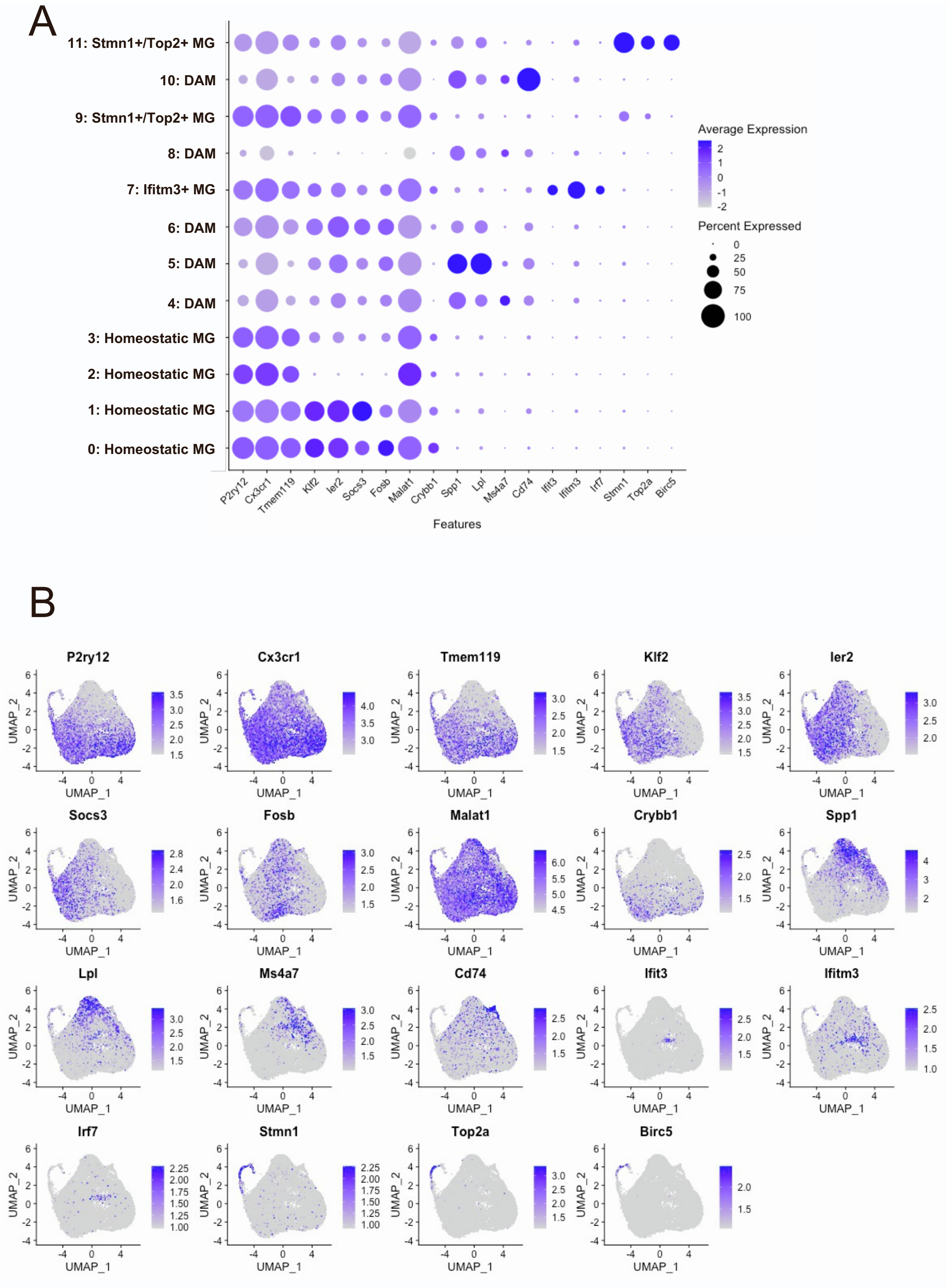
A
11: Stmn1+/Top2+ MG
10: DAM
9: Stmn1+/Top2+ MG
8: DAM
7: Ifitm3+ MG
6: DAM
5: DAM
4: DAM
3: Homeostatic MG
2: Homeostatic MG
1: Homeostatic MG
0: Homeostatic MG
P2ry12
Cx3cr1
Tmem119
Klf2
Ier2
Socs3
Fosb
Malat1
Crybb1
Spp1
Lpl
Ms4a7
Cd74
Ifit3
Ifitm3
Irf7
Stmn1
Top2a
Birc5
Features
Average Expression
Percent Expressed
B
P2ry12
Cx3cr1
Tmem119
Klf2
Ier2
Socs3
Fosb
Malat1
Crybb1
Spp1
Lpl
Ms4a7
Cd74
Ifit3
Ifitm3
Irf7
Stmn1
Top2a
Birc5
UMAP_1
UMAP_2

Figure S6. Characterization of each microglia cluster in 21,952 microglia from all strains, Related to Figure 6

(A) Dot plot showing averaged expression levels (color intensity) of microglia subtype marker genes and ratios of microglia expressing their genes (dot size) in each cluster. (B) UMAP plots showing expression levels of microglia subtype marker genes and distributions of microglia expressing their genes.

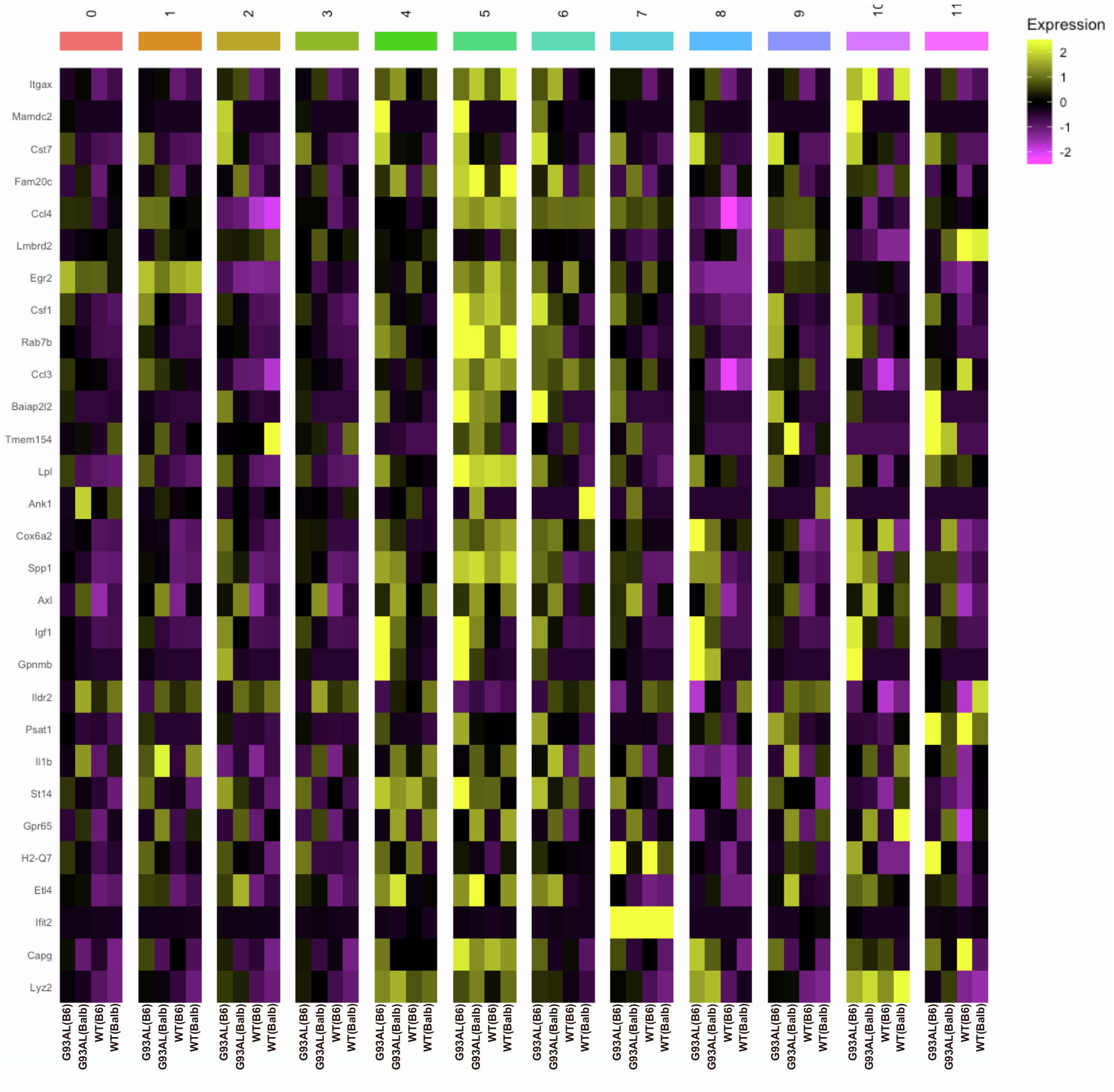

Figure S7. Heatmap of single-cell microglia transcriptome in DAM marker genes, Related to Figure 7

Heatmap showing expression levels of DAM marker genes in each cluster of single-cell microglia of each genotype.

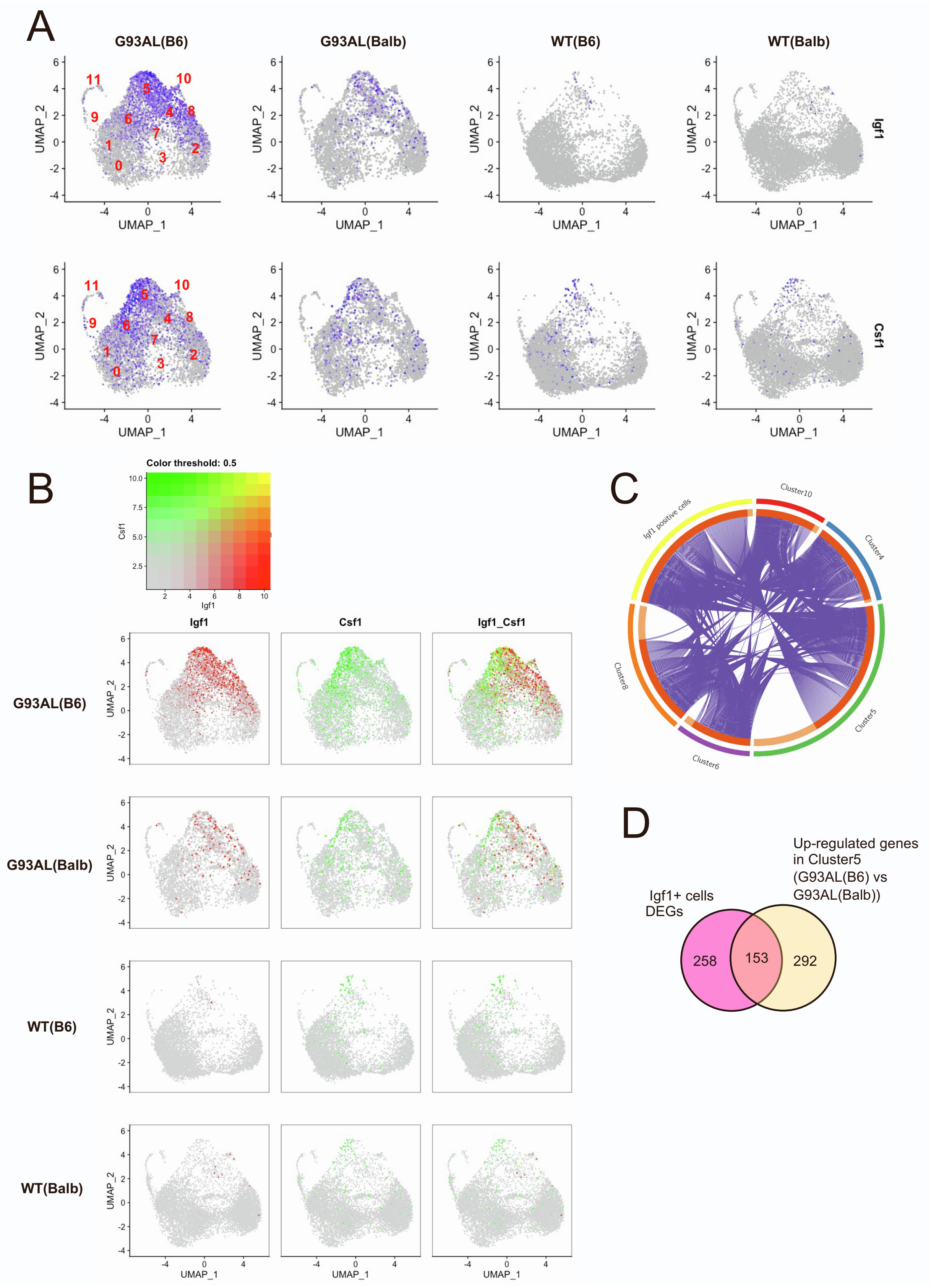

A
G93AL(B6)
G93AL(Balb)
WT(B6)
WT(Balb)
Igf1
Csf1
UMAP_1
UMAP_2
B
Color threshold: 0.5
Csf1
Igf1
Igf1_Csf1
G93AL(B6)
G93AL(Balb)
WT(B6)
WT(Balb)
C
Igf1 positive cells
Cluster10
Cluster4
Cluster5
Cluster6
Cluster8
D
Igf1+ cells DEGs
Up-regulated genes in Cluster5 (G93AL(B6) vs G93AL(Balb))
258
153
292

Figure S8. Gene expression analyses of single-cell microglia, Related to Figure 7

(A and B) UMAP plots showing expression levels of *Igf1* and *Csf1* and distributions of microglia expressing their genes. The numbers represent each microglia cluster. Merged UMAP plots were shown in (B). (C) Circos plot showing overlaps among the DEGs of each DAM cluster and *Igf1*-positive cells (microglia). (D) Venn diagram showing the comparison between DEGs of *Igf1*-positive cells (microglia) and upregulated genes in G93AL(B6) microglia cluster 5 relative to those in G93AL(Balb) microglia. The numbers represent gene numbers.

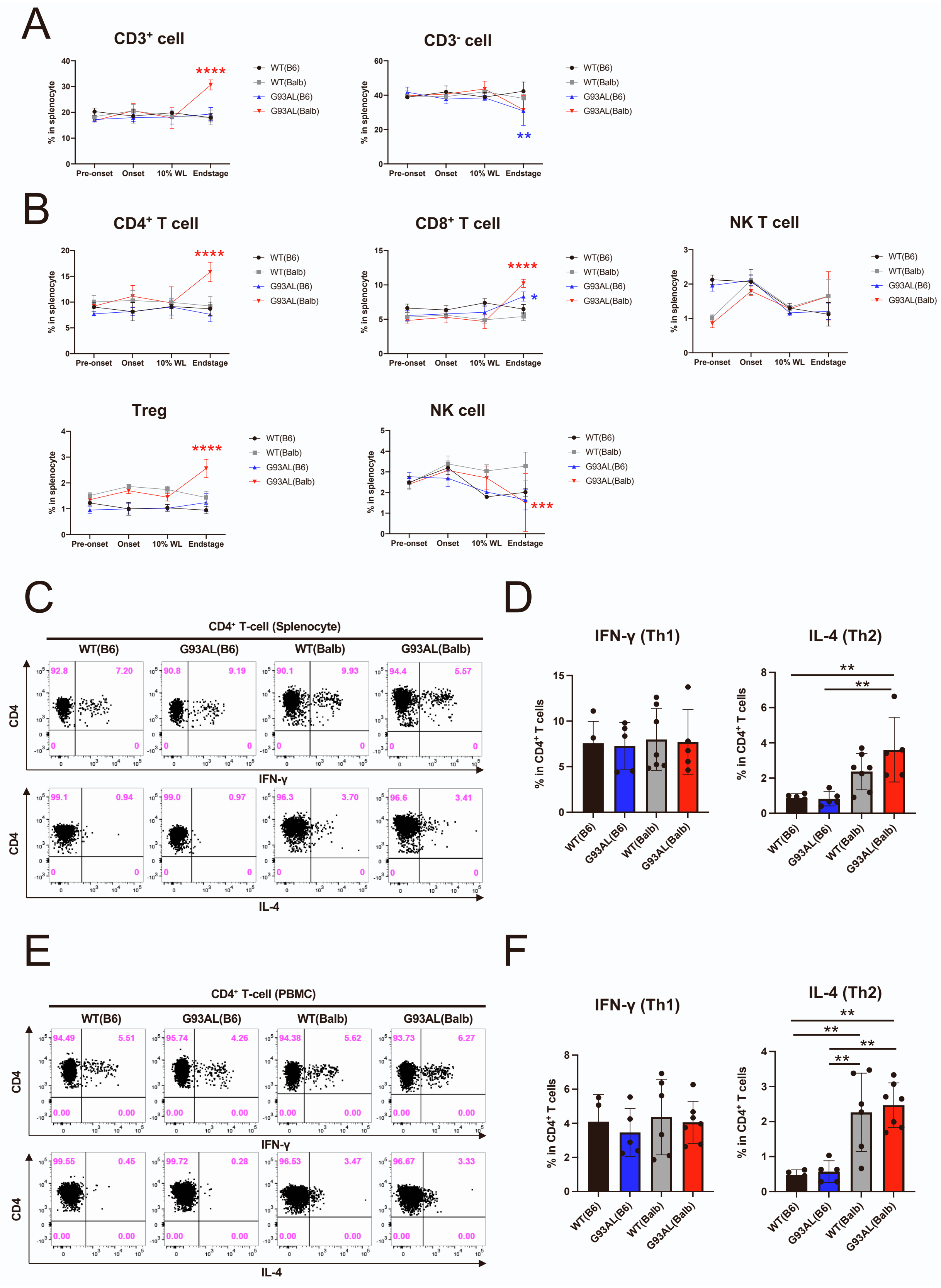
A
CD3+ cell
CD3- cell
% in splenocyte
Pre-onset
Onset
10% WL
Endstage
WT(B6)
WT(Balb)
G93AL(B6)
G93AL(Balb)
B
CD4+ T cell
CD8+ T cell
NK T cell
Treg
NK cell
C
CD4+ T-cell (Splenocyte)
WT(B6)
G93AL(B6)
WT(Balb)
G93AL(Balb)
CD4
IFN-γ
IL-4
D
IFN-γ (Th1)
IL-4 (Th2)
% in CD4+ T cells
E
CD4+ T-cell (PBMC)
F

Figure S9. Analyses of peripheral immune cells of mice with each genotype, Related to Figure 7

(A and B) Percentages of $CD45^{high}/CD3^{+}$ cells, $CD45^{high}/CD3^{-}$ cells, and subsets of immune cells in splenocytes were quantified by flow cytometric analysis at pre-onset (130 days of age (dA)), onset (145 dA), 10% body weight loss (10%WL) (WT(B6) and G93AL(B6): 200 dA, WT(Balb) and G93AL(Balb): 190 dA), and disease end-stage (pre-onset, 10%WL, and end-stage: n = 3 each; onset: WT(B6), G93AL(B6), and WT(Balb), n = 3, each; G93AL(Balb), n = 5). Data represented as mean ± SD. Two-way ANOVA followed by Tukey–Kramer multiple comparison post hoc tests at each time point. *$p < 0.05$, **$p < 0.01$, ***$p < 0.001$, ****$p < 0.0001$; G93AL(B6) vs WT(B6) as indicated by blue color, G93AL(Balb) vs WT(Balb) as indicated by red color. (C and D) Representative data of flow cytometric analysis of intracellular cytokine staining in splenocytes from each genotype (C). Percentages of IFN-g-producing $CD4^{+}$ T cells (Th1) and IL-4-producing ones (Th2) were plotted in (D) (WT(B6), G93AL(B6), and G93AL(Balb): n = 5, each; WT(Balb): n = 7). Data were represented as mean ± SD. One-way ANOVA followed by Tukey–Kramer multiple comparison post hoc test. **$p < 0.01$. (E and F) Representative data of flow cytometric analysis of intracellular cytokine staining in PBMCs from each genotype (E). Percentages of IFN-g-producing $CD4^{+}$ T cells (Th1) and IL-4-producing ones (Th2) were plotted in (F) (WT(B6): n = 4; G93AL(B6), n = 5; WT(Balb): n = 6; G93AL(Balb): n = 7). Data were represented as mean ± SD. One-way ANOVA followed by Tukey–Kramer multiple comparison post hoc test. **$p < 0.01$.

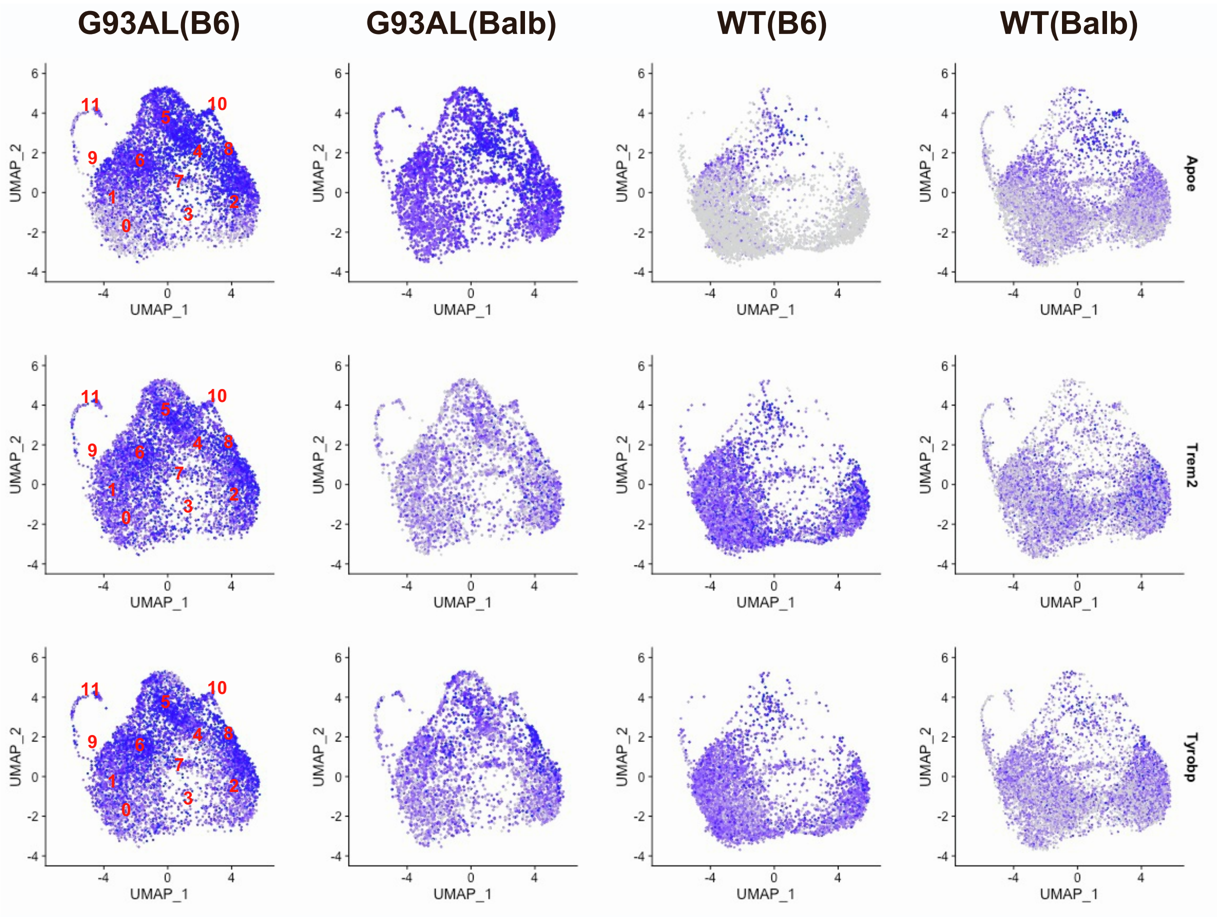

Figure S10. Gene expression analyses of *Apoe*, *Trem2*, and *Tyrobp*, Related to Figure 7

UMAP plots showing expression levels of *Apoe*, *Trem2*, and *Tyrobp*, and distributions of microglia expressing their genes. The numbers represent each microglia cluster.

Table S1. Identified marker genes characterizing each microglia cluster, Related to Figure 6 and Figure S6

Identities	Cluster No.	Marker genes			
Homeostatic MG	Cluster1	*Klf2(high)/Ier2(high)*	*Socs3(high)*	*Fosb(low)*	*Crybb1(low)*
	Cluster0			*Fosb(high)*	*Crybb1(high)*
	Cluster3	*Klf2(low)/Ier2(low)*	*Socs3(low)*	*Fosb(low)*	*Crybb1(low)*
	Cluster2	*Klf2-/Ier2-*	*Socs3-*		
DAM	Cluster5	*Spp1(high)*	*Lpl (high)*		
	Cluster4	*Spp1+*	*Lpl (low)*	*Klf2(low)/Ier2(low)*	*Ms4a7+*
	Cluster6				
	Cluster8			*Klf2-/Ier-*	*Socs3-/Fosb-/Malat1(low)*
	Cluster10			*Klf2(low)/Ier2(low)*	*Cd74+*
Ifitm3+ MG	Cluster7	*Ifit3+/Ifitm3+/Irf7+*			
Stmn1+/Top2+ MG	Cluster9	*Stmn1(low)/Top2a(low)*	*Birc5-*		
Stmn1+/Top2+ MG	Cluster11	*Stmn1(high)/Top2a(high)*	*Birc5+*		

Table S2. Representative gene lists from gene enrichment analysis of the common 153 genes between the DEGs of *Igf1*-positive microglia and upregulated genes in cluster 5, Related to Figure 7 and Figure S8D

Term	Description	LogP	Log(q-value)	Symbols
GO:0050866	negative regulation of cell activation	-6.1338758	-3.599	Apoe,Cd9,Cebpb,Cst7,Cd74,Lgals3,Serpine2,Tnfaip3,Tyrobp,Trem2,Gpnmb,Ctsl,Flt1,Igf1,Npm1,Timp2,Rps3,Chst2
GO:1901214	regulation of neuron death	-6.2822608	-3.731	Apoe,Cebpb,Csf1,Ddit3,Rack1,Gpi1,Igf1,Npm1,Ccl3,Tyrobp,Ubb,Ctsz,Trem2,Gpnmb
GO:0034341	response to type II interferon	-4.3109305	-2.065	Capg,Gapdh,Cd74,Ccl3,Ccl6,Rpl13a,Bst2,Csf1,Fcer1g,Flt1,Lgals3,Cyba,Ifi204,Mif,Trem2
GO:0150079	negative regulation of neuroinflammatory response	-3.8336077	-1.703	Cst7,Igf1,Trem2,Tnfaip3,Gpnmb,Ddit3,Flt1,Tyrobp,Bst2,Gnas,Naca,Serpine2,Rap2b,Rack1,Gpi1,Fabp5,Rps6,Apoe,Anxa3,Csf1,Cd74,Lgals3

Table S3. A list of primers used for qRT-PCR, Related to Figure 3A and 3E, Figure 4A, and Figure S2B

β-actin-f_1	ACAGCTTCTTTGCAGCTCCT
β-actin-r_1	ATTCCCACCATCACACCCTG
β-actin-f_2	TTGGCCTCACTGTCCACCTT
β-actin-r_2	CGGACTCATCGTACTCCTGCTT
Csf1-f	AGTATTGCCAAGGAGGTGTCAG
Csf-1-r	ATCTGGCATGAAGTCTCCATTT
Il34-f	CTTTGGGAAACGAGAATTTGGAGA
Il34-r	GCAATCCTGTAGTTGATGGGGAAG
Csf2-f	GGCCTTGGAAGCATGTAGAGG
Csf2-r	GGAGAACTCGTTAGAGACGACTT
Csf1r-f	GGACCTACCGTTGTACCGAG
Csf1r-r	CAAGAGTGGGCCGGATCTTT
Cd86-f	TTACGGAAGCACCCACGA
Cd86-r	GTAAATGGGCACGGCAGA
Ccl5-f	GCTGCTTTGCCTACCTCTCC
Ccl5-r	TCGAGTGACAAACACGACTGC
Cxcl10-f	GATGACGGGCCAGTGAGAA
Cxcl10-r	GCTCGCAGGGATGATTTCAA
Tnf-f	CCCTCACACTCAGATCATCTTCT
Tnf-r	GCTACGACGTGGGCTACAG
Igf1-f	CTGGACCAGAGACCCTTTG
Igf1-r	CCTGTGGGCTTGTTGAAGTAAAA
Mrc1-f	GCTGAATCCCAGAAATTCCGC
Mrc1-r	ATCACAGGCATACAGGGTGAC
Tgfb1-f	GCAGTGGCTGAACCAAGGA
Tgfb1-r	AGCAGTGAGCGCTGAATCG
Itgax-f	CTGGATAGCCTTTCTTCTGCTG
Itgax-r	GCACACTGTGTCCGAACTCA
Cd68-f	TGCGGCTCCCTGTGTGT
Cd68-r	TCTTCCTCTGTTCCTTGGGCTAT
Lgals3-f	CACGAAGCAGGACAATAACTGG
Lgals3-r	ATCGTTGACCGCAACCTTGAA
Aif1-f	ATTTGCAGGGAGGAAAAGCTT
Aif1-r	TGATCCCCTCCAGCCTCTCT
Gfap-f	TGACCGCTTTGCTAGCTACATC
Gfap-r	CCAGCGCCTTGTTTTGCT
